# Oriented Crystallization of Perovskite Film via Fluorine‐Containing Hyperbranched Polymer for Efficient and Stable Perovskite Solar Cells

**DOI:** 10.1002/adma.202511684

**Published:** 2025-09-18

**Authors:** Junyi Huang, Xiongjie Li, Zhiguo Zhang, Tianyu Sun, Hongliang Dong, Haixuan Yu, Xiaoting Ma, Wanpeng Yang, Letian Dai, Lei Wang, Bing Hu, Yan Shen, Mohammad Khaja Nazeeruddin, Mingkui Wang

**Affiliations:** ^1^ Wuhan National Laboratory for Optoelectronics Huazhong University of Science and Technology 1037 Luoyu Road Wuhan Hubei 430074 P. R. China; ^2^ Center for High‐Pressure Science and Technology Advanced Research Pudong Shanghai 201203 P. R. China; ^3^ Wuhan Hero Optoelectronics Technology Co., LTD 6 Huanglongshan North Road, East Lake High‐Tech Development Zone Wuhan Hubei 226514 P. R. China; ^4^ Institut des Sciences et Ingénierie Chimiques Ecole Polytechnique Fédérale de Lausanne Lausanne 1015 Switzerland; ^5^ Mechanical and Energy Engineering Department College of Engineering Imam Abdulrahman Bin Faisal University Dammam 34212 Saudi Arabia; ^6^ Optics Valley Laboratory Wuhan Hubei 430074 P. R. China

**Keywords:** additive, crystallization, hydrophobic effect, perovskite solar cell, stability

## Abstract

Solution‐processed perovskite solar cells have significant potential for large‐scale manufacture, but the production of perovskite film with high crystallinity over large areas remains a major challenge. Here, a fluorine‐containing hyperbranched polymer is shown for meticulous control of the perovskite film crystallization. Synergistic coordination of functional fluorine groups and perovskite species constrains the complex intermediate phases and facilitates the formation of spatially oriented perovskite films with high crystallinity and phase purity. Simultaneously, the thermal radical polymerization during the annealing process creates a cross‐linked hydrophobic network, which enhances resistance to moisture. This results in efficient regular planar perovskite solar cells with a remarkable power conversion efficiency of 26.05% for small devices (active area 0.04 cm^2^) and 22.43% for large devices (active area 16.1 cm^2^) under simulated AM 1.5G sunlight (100 mW cm^−2^). Moreover, the unencapsulated devices exhibit excellent operating stability, with 97% of initial efficiency remaining at the maximum power point tracking for 1500 h under continuous illumination (one sunlight intensity) at 50–55 °C.

## Graphical abstract

A fluorine‐containing hyperbranched polymer is developed to guide oriented crystallization and construct a cross‐linked hydrophobic network, enabling the formation of high‐quality perovskite films with enhanced environmental stability. The resulting devices deliver a PCE of 26.05% (0.04 cm^2^) and 22.43% (16.1 cm^2^), while maintaining 97% of their initial efficiency after 1500 h of unencapsulated operation at 50–55 °C under continuous illumination.

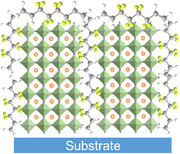

## 1 Introduction

Perovskite solar cells (PSCs), especially formamidinium (FA, CH(NH_2_)_2_^+^)‐based perovskites, have attracted attention due to high power conversion efficiencies (PCE) above 26%.^[^ ^1^, ^2^ ^]^ However, the photoactive black phase (*α*‐phase) of FA‐based perovskites is energetically unfavorable at temperatures below 150 °C, leading to the formation of yellow polytype (*δ*‐phase, i.e., 2H, 6H) and non‐photoactive intermediate phases during film deposition.^[^ ^3^, ^4^, ^5^ ^]^ The formation of solvated intermediates can be attributed to the polar aprotic solvent such as dimethyl sulfoxide (DMSO), which acts as a Lewis base and strongly coordinates with perovskite species.^[^ ^6^, ^7^ ^]^ The long‐term presence of residual phases leads to disordered growth of perovskite crystals. This prevents the proper orientation of the perovskite crystals, increases the defect density, and reduces charge carrier transport, ultimately resulting in low crystallinity *α*‐phase films with inferior optoelectronic properties.^[^ ^8^, ^9^, ^10^ ^]^ Therefore, the development of large‐area, high‐efficiency PSCs critically depends on the control over the crystallization process to achieve uniform, high‐purity *α*‐phase FA‐based perovskite films.

A methodology to produce pure and high‐quality *α*‐phase FA‐based perovskite films involves the incorporation of heterogeneous perovskite seeds into the precursor.^[^ ^11^, ^12^ ^]^ For example, the *δ*‐APbI_3_ (A = FA, MA, Cs, or Rb) single crystal serves as an effective template for the crystal growth pathways that form high‐quality perovskite films.^[^ ^13^, ^14^, ^15^ ^]^ An alternative methodology involves the incorporation of additives comprising organic ammonium salts or ionic liquids in the precursor.^[^ ^16^, ^17^, ^18^ ^]^ The presence of these additives has been demonstrated to result in the formation of new intermediate phases, thereby effectively hindering the interaction with solvent molecules while concurrently promoting ordered growth of perovskites. Of particular interest are additives containing fluorine (F), which have been examined in detail for their high electronegativity, hydrophobicity, and chemical stability.^[^ ^19^, ^20^ ^]^ The strong electronegativity of fluorine allows the additive to interact with perovskite precursors, modulating crystallization kinetics and suppressing the formation of non‐photoactive intermediate phases. Theoretical studies have shown that fluorine can form hydrogen bonds or coordinate with FA^+^ and Pb^2+^, thereby reducing defect densities and enhancing charge transport properties.^[^ ^21^, ^22^ ^]^ Notwithstanding these advancements, a number of reports indicate that the long‐term stability of devices containing an additive layer with a fluorine compound still requires improvement.^[^ ^23^ ^]^ For example, the small molecule fluorinated additives are unable to significantly improve the hydrophobicity of the perovskite surface.^[^ ^24^, ^25^ ^]^ Robust waterproofing is essential for the continued operational stability of photovoltaic devices, which must withstand a wide range of environmental conditions, including fluctuating humidity, temperature changes, and direct exposure to rain.^[^ ^26^ ^]^

This study investigates the microscale mechanism underlying the phase evolution of the perovskite film during crystallization, using the widely studied FA_0.9_MA_0.05_Cs_0.05_PbI_3_ perovskite film as a model. A fluorine‐containing branched polymer was developed to control the crystallization of the perovskite films. The fluorine‐containing branched polymer can be obtained by polymerizing a versatile organic monomer of 1,4‐divinyloctafluorobutane (DVFB), which contains functional fluorine groups and radically polymerizable double bonds. The fluorine groups of the DVFB synergistically coordinate with perovskite species, preventing the formation of solvent intermediate phases and promoting the growth of highly crystalline, phase‐pure perovskite films. The presence of an initiator and elevated temperatures facilitates polymerization via the radical double bond in DVFB, resulting in perovskite films with improved hydrophobicity. Simultaneously, the incorporation of reactive vinyl groups facilitates an additional cross‐linking process, thereby promoting the formation of large grains with a preferred orientation through the consumption of neighboring small, unaligned crystals.^[^ ^27^ ^]^

## 2 Results and Discussion

**Figure 1:**
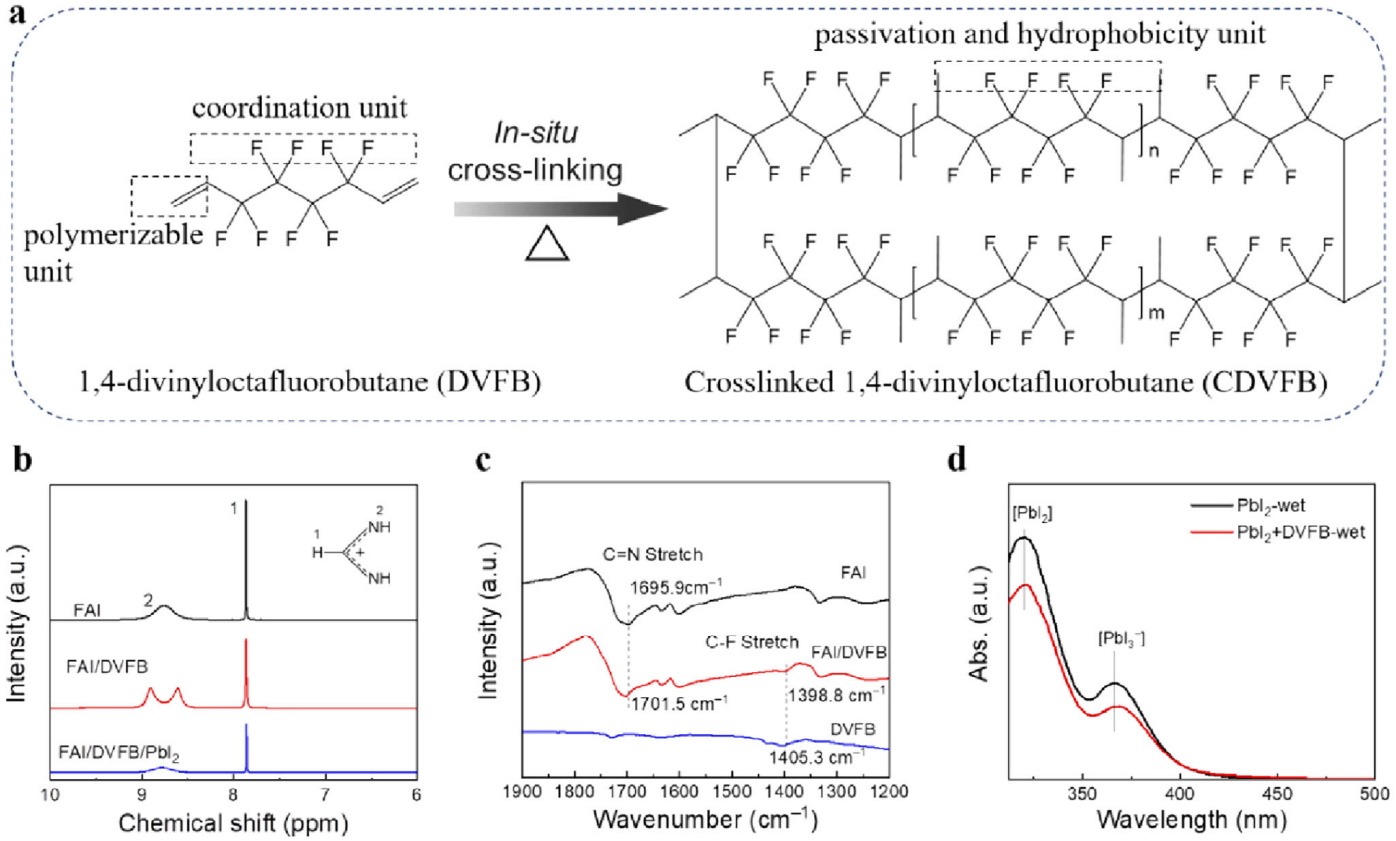
Synergistic coordination effect of DVFB with perovskites. a) Schematic diagram of the molecular structure and in situ cross‐linking polymerization of 1,4‐divinyloctafluorobutane (DVFB). b) A comparison of ^1^H NMR spectra of FAI (CH(NH_2_)_2_^+^I^−^), FAI+DVFB, and FAI+DVFB+PbI_2_. The measurements were performed in DMSO‐d_6_. c) A comparison of FTIR spectra of the FAI, DVFB, and FAI+DVFB. d) A comparison of UV–vis spectra of the wet films obtained by spin‐coating PbI_2_ and PbI_2_+DVFB in DMF solution.

**Figure** **1**a shows the molecular structure of DVFB as an additive of perovskite precursor, which is a di‐terminal alkene with polyfluorinated functional groups. The chemical can be synthesized by the addition of free radicals followed by dehydrohalogenation (Figure S1, Supporting Information).^[^ ^28^ ^]^ Figures S2 and S3 (Supporting Information) show the ^1^H NMR, ^13^C NMR, and mass spectra of DVFB, along with peak assignments. The DVFB monomer forms a cross‐linked DVFB polymer (CDVFB) at temperatures below 150 °C in the presence of azobisisobutyronitrile (AIBN) as initiator (0.01 mol%).^[^ ^29^ ^]^ The initiator decomposes rapidly to generate free radicals that break the C═C bond of the terminal alkenes and are subsequently recombined with the adjacent monomeric units (Figure S4, Supporting Information).^[^ ^29^ ^]^ The small amount of AIBN decomposes completely during annealing, with no significant impact on the quality of the perovskite film or the performance of the resulting device (Figure S5, Supporting Information). Figure S6 (Supporting Information) shows photographs of DVFB with AIBN as the initiator before and after heating at 150°C for 10 min, showing the transition from liquid (monomeric) to transparent solid (polymer) state. Further Fourier transform infrared spectroscopy (FTIR) characterization was conducted to verify the cross‐linking of DVFB in the perovskite films. As shown in Figure S7 (Supporting Information), the C═C vibration peaks^[^ ^30^ ^]^ almost completely disappear after annealing at 150 °C for 10 min. After adding into the perovskite precursor (i.e., 0.01 mm DVFB, 1.3 mm PbI_2_, and 1.2 mm FAI in DMF/DMSO mixed solvents), the coordination between DVFB and PbI_2_ can be formed by sharing lone pair electrons in the F‐functional group with the empty orbitals of Pb^2+^ (Figure S8b, Supporting Information).^[^ ^31^ ^]^ At the same time, the high electronegativity of F induces a polarization of electron density in the adjacent N–H bonds, resulting in a localized accumulation of electrons around the F atom and the subsequent formation of N–H···F hydrogen bonds, with hydrogen acting as a proton donor and fluorine as an electron acceptor (Figure S8c, Supporting Information). Such an interaction can be verified by density functional theory (DFT) calculation. Figure S9 (Supporting Information) shows the assessed binding activity of DVFB to PbI_2_ or FAI (CH(NH_2_)_2_I). The binding energy for the DVFB·PbI_2_ and DVFB·FA^+^ (CH(NH_2_)_2_^+^) is estimated to be −1.76 and −1.40 eV, respectively, which is higher than the bending energy of DMSO·PbI_2_ (−0.88 eV). This descriptor indicates that DVFB·PbI_2_ and DVFB·FA^+^ are thermodynamically favored.^[^ ^30^, ^31^ ^]^

Liquid‐state proton nuclear magnetic resonance (^1^H NMR) analysis was carried out to investigate the interference of DVFB in solvent intermediate phases. As shown in Figure 1b, the resonance signal of protonated ammonium from FAI appears at 8.75 ppm in the pure deuterated DMSO solution. After adding DVFB, the resonance signal of ammonium is divided into 8.91 and 8.61 ppm. This reflects the local disturbance of the electronic interference of FA^+^ ammonium due to the interaction of N–H···F hydrogen bonds (Figure S8c, Supporting Information). The resonance signals of ammonium amalgamate are combined into a single peak at 8.79 ppm by adding PbI_2_, with a decrease in signal intensity, suggesting that the interaction between PbI_2_ and DVFB is more pronounced than that between FA^+^ and DVFB. This observation indicates a synergistic coordination where the DVFB interacts with the FA^+^ and PbI_2_ simultaneously, thereby weakening the coordination of the solvents.^[^ ^32^, ^33^ ^]^ This synergistic coordination thermodynamically breaks the network of coordination of solvents, thereby preventing the formation of the solvent intermediate phase.

The suppression of solvent intermediate phases in the presence of DVFB through synergistic coordination with FAI and PbI_2_ is further confirmed by FTIR, UV–vis spectroscopy, and X‐ray photoelectron spectroscopy (XPS). The FTIR spectra show that after adding DVFB to FAI, the C═N stretching vibration peak of the pure FAI blueshifts from 1695.9 to 1701.5 cm^−1^, and the C–F stretching vibration redshifts from 1405.3 cm^−1^ in DVFB to 1398.8 cm^−1^ (Figure 1c). This can be attributed to the formation of N–H···F hydrogen bond interaction as described in ^1^H NMR characterization.^[^ ^34^ ^]^ The UV–vis absorption analysis was performed on wet film prepared by spin‐coating PbI_2_ or PbI_2_ + DVFB in DMF/DMSO solution on a quartz substrate. For pure PbI_2_ wet film, the exact absorption edges of PbI_2_ and [PbI_3_]^−^ (starting at 319 and 366 nm, respectively) are observed in Figure 1d. After the addition of DVFB, redshift of absorption edges of PbI_2_ (321 nm) and [PbI_3_]^−^ (369 nm) is noted in the wet films. This is ascribed to the formation of adducts of DVFB·PbI_2_ and DVFB·[PbI_3_]^−^.^[^ ^35^ ^]^ The coordination interaction between DVFB and PbI_2_ was further verified with XPS characterization. As shown in Figure S10a (Supporting Information), the binding energy at 143.12 and 138.26 eV for Pb 4f5/2 and Pb 4f7/2 species of the perovskite without DVFB film shifted to lower values of 142.88 and 137.99 eV upon the introduction of DVFB. This result reflects the electron transfer from fluorine of DVFB to Pb^2+^ (Figure S8b, Supporting Information).^[^ ^36^ ^]^ In addition, Figure S10b (Supporting Information) shows the F 1s XPS spectra of pure DVFB and perovskite films with DVFB. After incorporation into the perovskite, the F 1s peak shifts to higher binding energy and becomes broader. This behavior is attributed to changes in the chemical environment of fluorine atoms caused by hydrogen bonding (N–H···F) and coordination with Pb^2+^ (Figure S8, Supporting Information), leading to a diverse distribution of local electronic environments and further confirming the interaction with the perovskite species.

**Figure 2:**
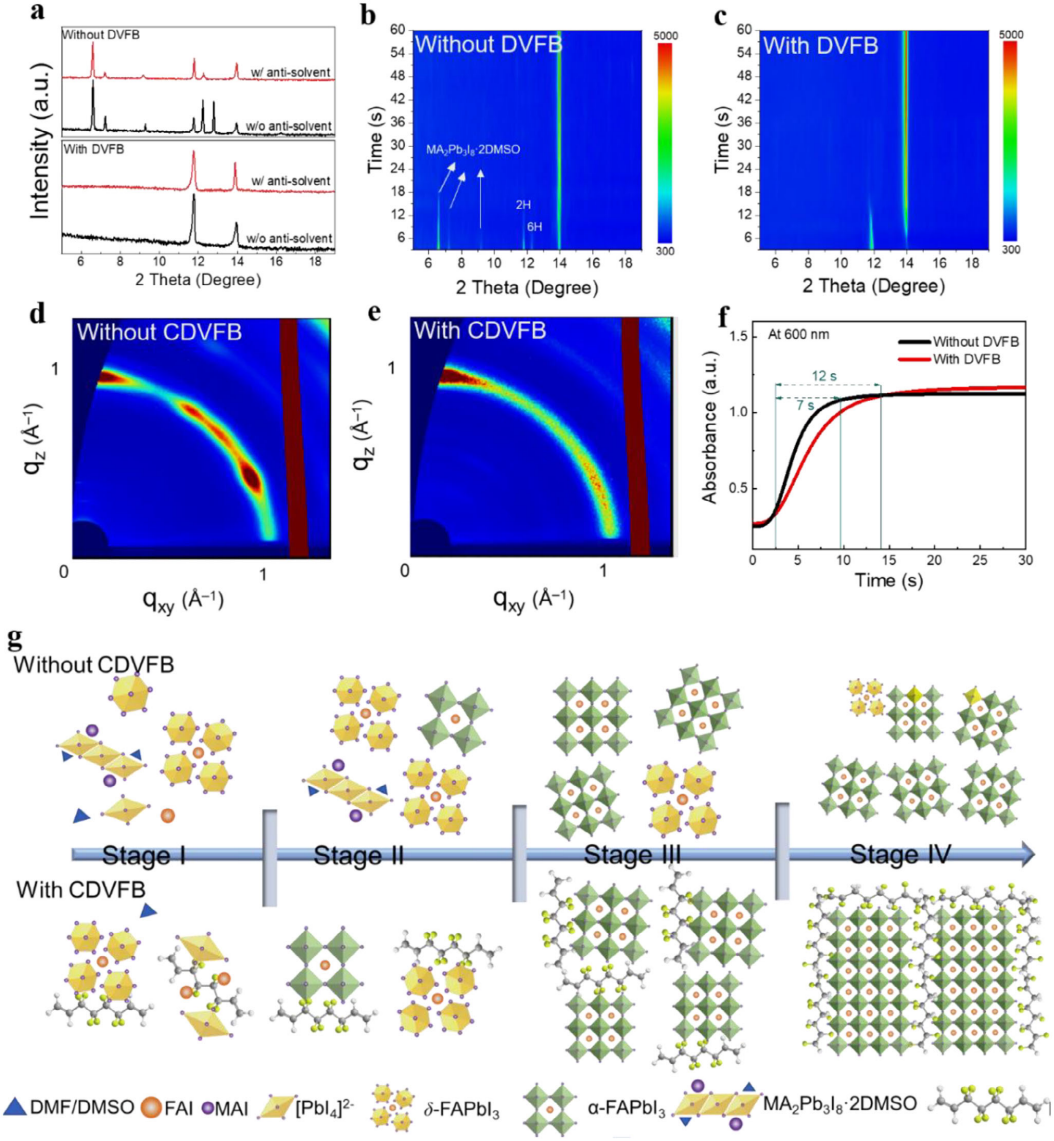
The effect of DVFB on intermediate phase, nucleation, and crystallization. a) XRD patterns of wet perovskite films obtained by spin‐coating the precursors with and without anti‐solution at room temperature. In situ XRD patterns of perovskite films fabricated b) without and c) with DVFB during the initial annealing process at 100 °C. GIWAX patterns of the perovskite film without d) and with e) CDVFB after annealed at 100 °C for 1 h and at 150 °C for 10 min. f) The corresponding absorption intensity changes at 600 nm for the perovskite film with and without CDVFB, extracted from the in situ absorption spectra (Figure S9, Supporting Information). g) Schematic diagram of the possible phase evolution of the nucleation and crystallization of FA‐based mixed cation perovskites during the film‐forming process without and with CDVFB.

**Figure** **2**a shows XRD patterns of wet perovskite films (i.e., prior to the annealing process) treated with or without antisolvent. In the pure perovskite wet films without antisolvent treatment, the predominant formation is a mixture of solvate interphase complexes (2θ = 6.60°, 7.23°, 9.27°) and hexagonal perovskite polytype interphases complexes 2H (2θ = 11.77°) and 6H (2θ = 12.23°), with a minimal presence of the *α*‐phase perovskite (2θ = 13.95°).^[^ ^22^ ^]^ For the same wet film treated with chlorobenzene antisolvent, the partial solubility intermediate phase (2θ = 6.60°, 7.23°, 9.27°) and 6H intermediate phase (2θ = 12.23°) transformed to the *α*‐phase (2θ = 13.95°) perovskite and 2H intermediate phases (2θ = 11.77°). Notably, in the DVFB‐treated perovskite wet films without antisolvent, the XRD pattern exhibits a dominant 2H intermediate phase (2θ = 11.77°) and an *α*‐phase perovskite (2θ = 13.95°), with a virtually disappeared intensity of complex solvate interphases (2θ = 6.60°, 7.23°, 9.27°) and hexagonal polytypes (6H phase at 12.23°). This explains the synergistic effect of the DVFB in disrupting the solvent‐related coordination networks and thus in suppressing complex intermediates. When the antisolvent is applied to films with CDVFB, the residual hexagonal 2H intermediate phase (2θ = 11.77°) is directly converted into the *α*‐phase perovskite (2θ = 13.95°).

We also monitored the XRD patterns of the perovskite film during the annealing stage in order to track the crystallization process. For the perovskite films without CDVFB (Figure 2b), the peak area of the mixed intermediate phases (6° to 13°) corresponds to their crystalline domain population. Along with the rapid decline of the peak area for the mixed intermediate phases, the peak area for the *α*‐phase perovskite (2θ = 13.95°) increases rapidly and reaches a plateau after ≈30 s. For the perovskite films with DVFB (Figure 2c), the solvate interphase (2θ = 6.60°, 7.23°, 9.27°) and the 6H interphase (2θ = 12.23°) are eliminated. Consequently, the 2H interphase (2θ = 11.77°) is transformed into the *α*‐phase perovskite (2θ = 13.95°). The peak area of the 2H phase (2θ = 11.77°) decreased to zero after 15 s, whereas the peak area of the *α*‐phase perovskite (2θ = 13.95°) increased further and remained stable until 40 s. Therefore, the incorporation of DVFB changes the conventional conversion pathways from solvate phase+2H phase+6H phase→*α* phase to 2H phase→*α* phase (Figure S11, Supporting Information). After 10 min of annealing at 150 °C, the XRD peaks corresponding to the PbI_2_ and 2H phases are visible in the pristine film, with the XRD patterns dominated by the *α*‐phase perovskite peak (Figure S12, Supporting Information). The film with CDVFB exhibits enhanced diffraction peaks of single *α*‐phase perovskite, and the undesired signal of the PbI_2_ and 2H phases disappears. Furthermore, the film with CDVFB shows a significant improvement in the ratio of the peak intensity (13.6) between the crystal planes (100) and (111) compared to the pristine film (5.8). This further confirms that the solvent intermediate phase‐dominated nucleation produces irregular grains and that incorporation of CDVFB therefore promotes (100)‐oriented crystallization.^[^ ^37^ ^]^ Additionally, the XRD patterns of the perovskite films with and without DVFB show no peak shift, and both films exhibit a consistent optical bandgap of 1.54 eV (Figure S13, Supporting Information). These results confirm that DVFB does not alter the perovskite lattice structure or its bandgap.

The grazing incidence wide‐angle X‐ray scattering (GIWAXS) characterization was further performed to investigate the crystal orientation of perovskite films after annealing. The azimuthal integration of crystal planes (100) of a pure perovskite film shows a wide distribution with peaks at different angles, indicating a random crystallographic orientation (Figure 2d). In contrast, only one sharp peak centered at a low azimuthal angle is observed for the perovskite film treated with CDVFB (Figure 2e). The low azimuthal angle indicates a higher preference for out‐of‐plane orientation perpendicular to the film plane.^[^ ^33^ ^]^ Consequently, the extracted azimuthal intensity profile shows a concentration at low angles (Figure S14, Supporting Information), thereby suggesting that the perovskite film with CDVFB exhibits a higher degree of out‐of‐plane orientation along the (100) facet. This orientation is known to facilitate vertical transport of charge carriers in perovskite films.^[^ ^33^ ^]^ Furthermore, the results of expanded GIWAXS measurements further confirmed improved phase purity and enhanced out‐of‐plane orientation in the perovskite film with CDVFB (Figure S15, Supporting Information). These observations suggest that perovskite treated with CDVFB can effectively circumvent the formation of undesired intermediates by modulating the nucleation dynamics and thereby reducing the nucleation pathways. This leads to a more controlled and preferential (100)‐oriented crystallization, ultimately contributing to the formation of high‐quality perovskite films with enhanced optoelectronic properties.

The nucleation process was monitored by in situ photoluminescence (PL) during the spin‐coating process. As shown in Figure S16 (Supporting Information), a PL signal at ≈760 nm appeared immediately after the anti‐solvent was dropped, which is characteristic of the intermediate phase.^[^ ^38^, ^39^ ^]^ The sample with DVFB exhibited a slower PL intensity increase compared to the pristine film, indicating a delayed and more controlled nucleation behavior. Figure 2f shows the change in the corresponding absorption intensity of these films at 600 nm versus the annealing time, which corresponds to a strong absorption region of the *α*‐phase perovskite. The absorbance was extracted from the UV–vis absorption spectra results (Figure S17, Supporting Information). Perovskite film treated with DVFB exhibits a longer time to complete crystallization (≈12 s) than the film without (≈7 s). This indicates a delayed crystallization dynamics in the perovskite films treated with DVFB. The extended crystallization is ascribed to an increased energy barrier for perovskite nucleation, which can be attributed to the synergistic coordination of DVFB with FAI and PbI_2_.^[^ ^40^ ^]^

On the basis of the aforementioned analysis, it can be concluded that DVFB is likely to play a regulatory role in the perovskite crystal pathway, as illustrated schematically in Figure 2g. Stage I: During the spin‐coating process, DVFB synergistically coordinates with FAI and PbI_2_ to form an adduct that kinetically blocks the solvent intermediate phase‐dominated nucleation. Stage II: Antisolvent treatment induces the adduct reorganization into a 2H‐DVFB·FAPbI_3_ framework, concurrently enabling direct 2H→*α*‐phase conversion. In contrast, the control films exhibit multipath phase transitions (solvate+2H+6H→*α*), which retain residual disordered intermediates. Stage III: During the early stage of the annealing process, the 2H‐DVFB·FAPbI_3_ template transforms into *α*‐phase DVFB·FAPbI_3_ with preferential (100)‐out‐of‐plane orientation. However, control films yield randomly oriented *α*‐phase grains with residual 2H defects. Stage IV: In situ DVFB cross‐linking during the prolonged annealing process further stabilizes the phase‐pure *α*‐FAPbI_3_ crystallization by consuming neighboring small, unaligned crystals.

**Figure 3:**
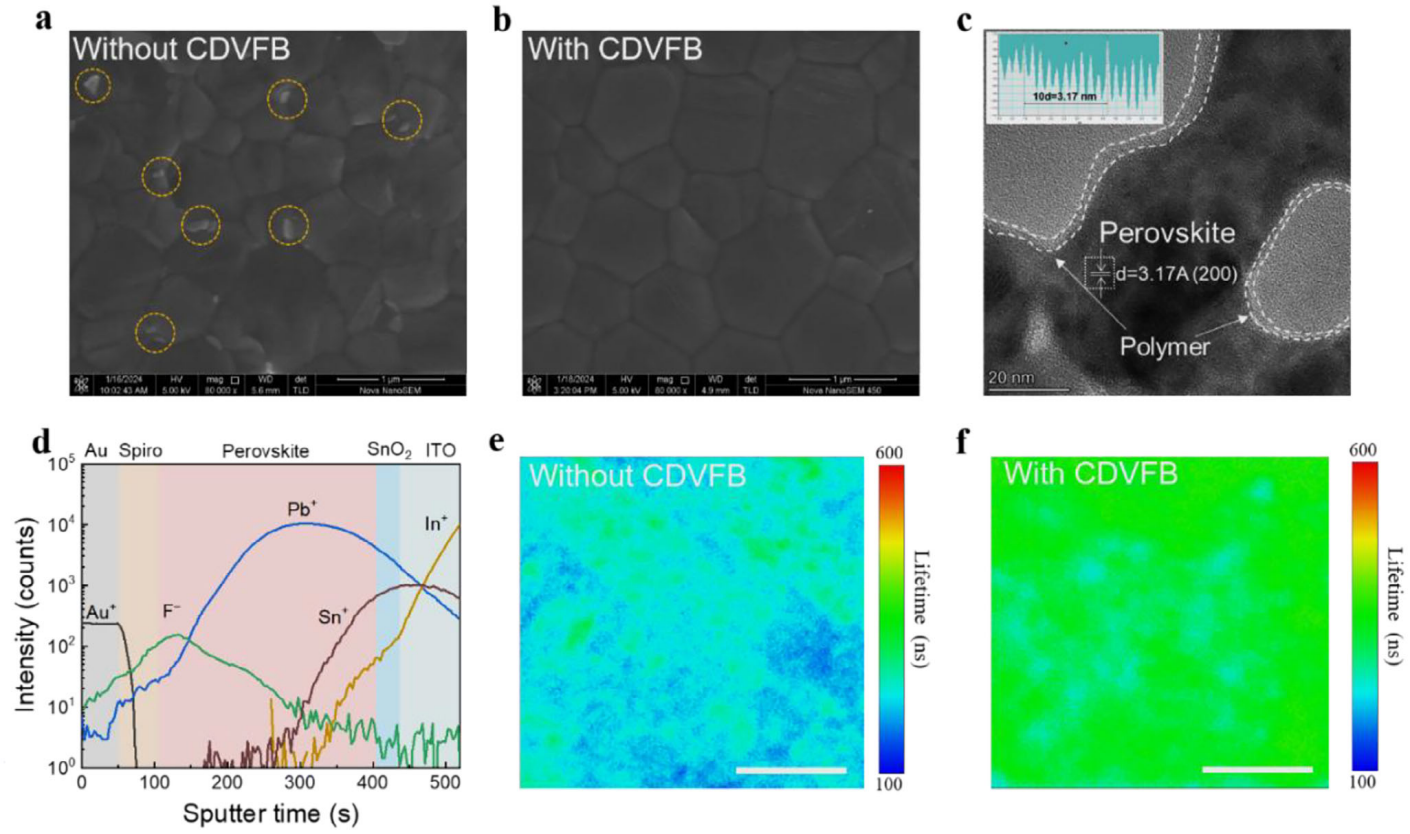
The effect of CDVFB on perovskite films. Top‐view SEM images of the perovskite film surface, a) without and with b) the CDVFB. c) TEM image clearly shows the CDVFB surround the grain boundaries of perovskite. The inset is the intensity profile recorded from the corresponding region shown in the panel. d) TOF‐SIMS spectra of the perovskite devices with CDVFB. Fluorescence lifetime imaging microscopy of the perovskite film surface e) without and f) with CDVFB. Scale bars, 10 µm.

As shown in **Figure** **3**a, scanning electron microscope (SEM) images of a perovskite film without CDVFB reveal the presence of a white, regular, and sharp‐edged substance, which is identified as PbI_2_.^[^ ^41^ ^]^ PbI_2_ is absent in the perovskite film with CDVFB (Figure 3b). At the same time, the average grain size of perovskite film with CDVFB increases significantly, almost doubling from ≈355 to 791 nm (Figure 3a,b; Figure S18, Supporting Information). The cross‐section morphology in Figure S19 (Supporting Information) further supports the fact that the perovskite film with CDVFB has a larger grain size and monolayer structure, which is advantageous for vertical carrier transport.^[^ ^42^ ^]^

The transmission electron microscopy (TEM) image in Figure 3c shows that the perovskite crystal regions have distinct lattice fringes. CDVFB is characterized by a recognizable amorphous region that is located either inside or along the edges of the crystal morphology.^[^ ^43^ ^]^ From the crystalline region, an interplanar spacing of 3.17 Å can be determined, corresponding well to the (100) plane of the cubic perovskite phase. Furthermore, the vertical distribution of CDVFB in perovskite films was analyzed by time‐of‐flight secondary ion mass spectrometry (ToF‐SIMS) characterization of the perovskite cells. As shown in Figure 3d, a substantial F signal of CDVFB was identified with a gradient distribution near the surface of the perovskite film, presumably arising from the migration of low‐surface‐energy CDVFB during the annealing process, driven by interfacial energy minimization.^[^ ^44^, ^45^ ^]^ As demonstrated by extant literature, the process has been shown to enhance surface passivation and moisture resistance, thus contributing to an improvement in device performance and stability.^[^ ^46^, ^47^ ^]^ Figure S20 (Supporting Information) shows the 2D ToF‐SIMS images of the distribution of F and Pb on the perovskite layer, which further supports the accumulation of CDVFB at the grain boundaries.

We examined the effect of CDVFB on the dynamics of charge recombination in perovskite films by means of PL and time‐resolved photoluminescence (TRPL) spectroscopy. The perovskite film treated with CDVFB shows a six‐fold increase in steady state PL intensity compared to the pristine film without a noticeable shift in peak positions (Figure S21, Supporting Information). Furthermore, the three‐fold increase in carrier lifetime of perovskite film with CDVFB (1176 ns) compared to pristine film (302 ns) suggests that the former is less prone to defects (Figure S22, Supporting Information). A further comparison of these composite characteristics by fluorescence lifetime imaging microscopy (Figure 3e,f), supported by statistical lifetime distribution histograms (Figure S23, Supporting Information), reveals that perovskite film treated with CDVFB consistently exhibits a 2 to 3‐fold increase in average PL lifetime and a significant increase in uniformity over the test area of 30 µm × 30 µm compared to the pristine perovskite film. This enhancement can be attributable to the modulation of the crystallization pathway and to the assurance of homogenous growth of the perovskite film, which is facilitated by the introduction of CDVFB.^[^ ^48^, ^49^ ^]^ The presence of a highly branched CDVFB on the grain boundaries of perovskite film has been shown to passivate defects and impede the penetration of water and oxygen (Figure S24, Supporting Information). It has been demonstrated that defect passivation, when employed in conjunction with enhanced crystallization, results in the formation of enlarged and more uniform grains. This, in turn, has been shown to enhance device performance and environmental stability.

**Figure 4:**
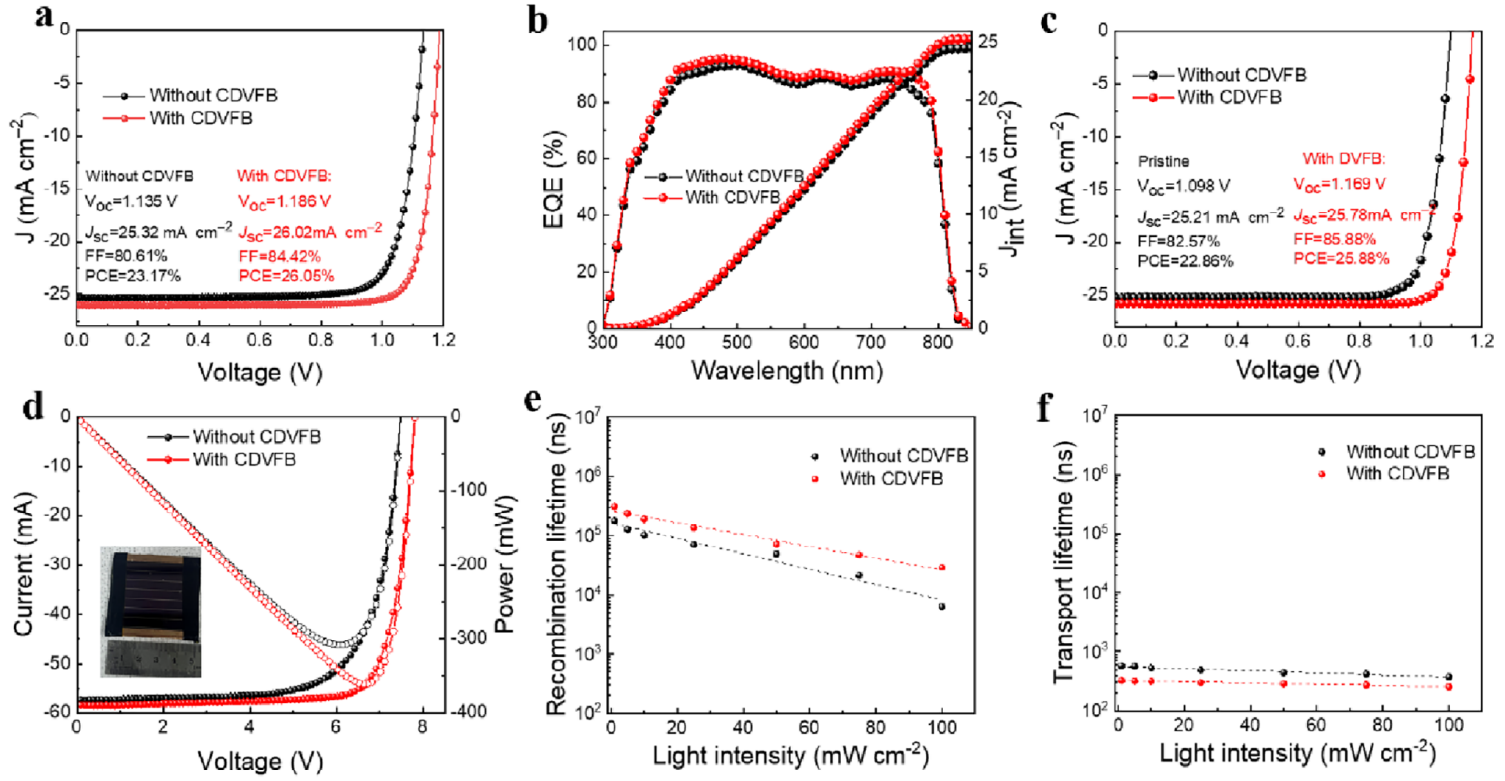
Effect of CDVFB on the performance of PSC devices. a) *J–V* curves of the best‐performing regular (n‐i‐p) devices using perovskite treated with and without CDVFB measured under AM 1.5 G illumination of 100 mW cm^−2^ in reverse scan. b) EQE spectra of the devices using perovskite treated with and without CDVFB. c) *J–V* curves of the best‐performing inverted (p‐i‐n) devices using perovskite treated with and without CDVFB measured under AM 1.5 G illumination of 100 mW cm^−2^ in reverse scan. d) *J–V* curves of the best‐performing large‐area regular module (16.1 cm^2^) using perovskite treated with and without CDVFB. The inset shows a photograph of a solar module. e) Recombination lifetime and f) charge transport lifetime of the devices using perovskite treated with and without CDVFB obtained by fitting the transient photocurrent/photovoltage decay curves.

Based on the above discussion, we designed a regular n‐i‐p perovskite device with an ITO/SnO_2_/Cs_0.05_MA_0.05_FA_0.9_PbI_3_/spiro‐OMeTAD/Au configuration to evaluate the influence of DVFB modification on photovoltaic performance (Figure S25, Supporting Information). The optimal concentration of DVFB in perovskite precursor solution was found to be 1.5 mg mL^−1^, resulting in a higher average PCE value (Figure S26, Supporting Information). The current density–voltage (*J*–*V*) curves of the lab cell (active area 0.04 cm^2^) with and without DVFB in the reverse scan (RS) under the AM 1.5G condition are presented in **Figure** **4**a. The PSCs without CDVFB treatment exhibited a PCE of 23.17%, in conjunction with a short‐circuit current density (*J*_SC_) of 25.32 mA cm^−2^, an open‐circuit voltage (*V*_OC_) of 1.135 V, and a fill factor (*FF*) of 81%. The devices with CDVFB treatment had undergone treatment showed a PCE of 26.05%, accompanied by a *V*_OC_ of 1.186 V, a *J*_SC_ of 26.02 mA cm^−2^, and a *FF* of 84%. The statistical distributions of four key photovoltaic parameters measured across 20 individual PSCs are presented in Figure S27 (Supporting Information). The corresponding *J*–*V* curve in the forward scan (FS) is presented in Figure S28 (Supporting Information). The increase in PCE can be attributed mainly to the augmented *V*_OC_ and *J*_SC_. In addition, the external quantum efficiency (EQE) has been determined and is shown in Figure 4b. The PSC device with CDVFB treatment exhibits a higher EQE response at longer wavelengths, indicating reduced surface recombination and long diffusion length. The integrated current density extracted from the EQE is 25.32 mA cm^−2^ for the CDVFB‐treated PSC, which is in line with the *J*–*V* measurements. To confirm the effectiveness and universality of CDVFB in enhancing efficiency, PSCs with an inverted (p‐i‐n) structure of ITO/NiO_X_/MeO‐2PACz/Cs_0.05_MA_0.05_FA_0.9_PbI_3_/ PCBM/BCP/Ag were also fabricated. The inverted PSCs with CDVFB treatment achieved a PCE of 25.88%, accompanied by a *J*_SC_ of 25.78 mA cm^−2^, a *V*_OC_ of 1.169 V, and a *FF* of 86% (Figure 4c). This performance metric is superior to that of the pristine device (22.86%). The corresponding EQE spectra are shown in Figure S29 (Supporting Information), thereby further corroborating the universality of enhanced photocurrent response. The enhanced efficiency can be attributed to the homogeneous growth of the perovskite film, which effectively reduces the defect state and mitigates non‐radiative recombination (Figure S30, Supporting Information).

In addition, the compatibility of the CDVFB with large‐scale fabrication of regular perovskite devices was tested by extending it to the production of large‐scale solar modules. For the perovskite module with CDVFB treatment composed of seven series‐connected sub‐cells with a surface area of 16.1 cm^2^, a high PCE of 22.43% (*V*_OC_ = 7.81 V, *J*_SC_ = 3.63 mA cm^−2^, FF = 79%) was obtained (Figure 4d). The detailed parameters of the device are also listed in Table S1 (Supporting Information). Furthermore, the perovskite modules treated with CDVFB (active area 16.1 cm^2^) show an average PCE of 22.21%, further demonstrating the applicability of this strategy (Figure S31, Supporting Information).

Transient photocurrent and photovoltaic decay (TPC and TPV) measurements were performed to investigate the transport and recombination processes in complete devices under different light intensities (Figure S32, Supporting Information). Figure 4e,f present the carrier recombination lifetime (τ_re_) and carrier transport lifetime (τ_tr_) as a function of the incident light intensity obtained by fitting the TPV/TPC curves for PSCs with and without CDVFB treatment. The device with CDVFB has a longer recombination lifetime than the device without treatment, indicating a lower probability of charge recombination. The carrier transport lifetime of the device with CDVFB is shorter than that of the control device, which results in a faster charge collection probability. Furthermore, the carrier diffusion coefficient (*D*), determined by *D*  = *d* ^2^/(*c* × τ_*tr*_)  (where *d* represents the absorber thickness and c is a constant), increases from 2.78 × 10^−3^ to 4.09 × 10^−3^ cm^2^ s^−1^ under 1 sunlight intensity for the device with CDVFB (Figure S33a, Supporting Information). The diffusion length (*L*) of the device can be described by the expression $L\;={\left(D\times \tau_{re}\right)}^{1/2}$. Accordingly, the device with CDVFB shows an increased diffusion length from 1323 to 3446 nm at 1 sunlight intensity (Figure S33b, Supporting Information), which indicates an efficient charge‐collecting capability.^[^ ^43^, ^49^ ^]^

**Figure 5:**
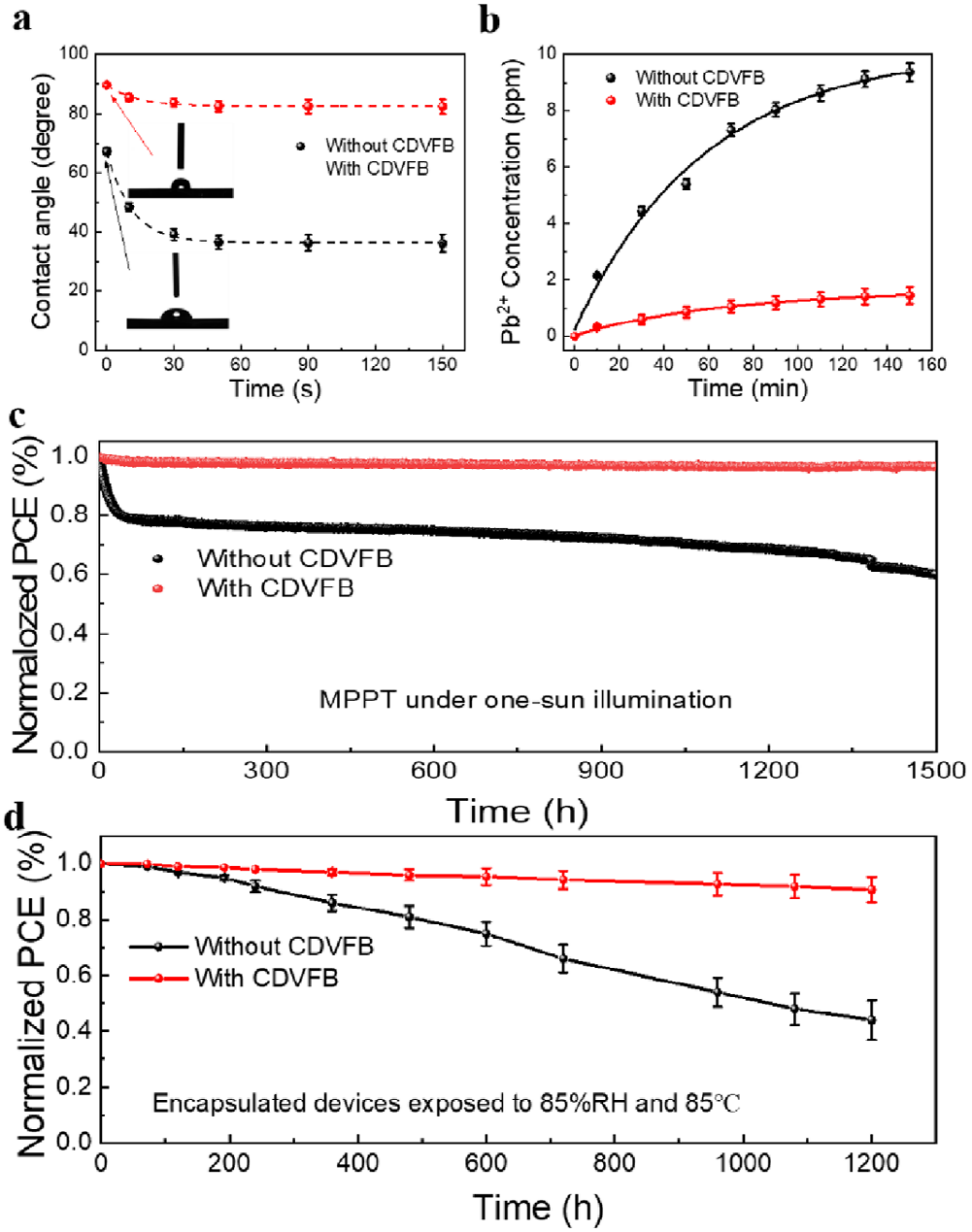
Effect of CDVFB on stability of PSC devices. a) Time‐dependent water contact angle measurement on perovskite films. The insets show the original contact angle of perovskite films treated with and without CDVBF. b) Time variation of lead concentration in water after immersing the device using perovskites treated with and without CDVFB at 30 °C. c) The aging test with MPPT tracking measurement of the unencapsulated devices using perovskites treated with and without CDVFB under full solar illumination (100 mW cm^−2^) at 50–55 °C. d) Stability measurements of encapsulated devices aged under 85 °C and 85% RH in the dark.

Time‐dependent contact angle measurements were conducted using a pendant water droplet method (5 µL deionized water) for the determination of the water resistance of the perovskite film with or without CDVFB. As shown in **Figure** **5**a, the water contact angle of the film without CDVFB decreases from 67.2° to 36.1° for 150 s, while the angle of the perovskite film treated with CDVFB remained at a high value of from 89.6° to 82.3°. The enhanced water resistance of films treated with CDVFB can be attributed to the hydrophobic effect of a highly branched polymer with a high number of fluorinated groups on the grain boundary and perovskite surface.^[^ ^50^ ^]^ This helps to maintain the stability of the perovskite film. Remarkably, the films with CDVFB retained their black phase of perovskite without any change in color even after prolonged immersion in water for over 100 s (Media S1, Supporting Information). This result confirms the effectiveness of CDVFB in impeding the penetration of H_2_O molecules from all directions. The formation of a protective barrier by a highly hydrophobic perovskite film has been shown to significantly reduce the risk of leakage of lead by reducing the infiltration of water.^[^ ^22^ ^]^ The unpackaged device was immersed in 50 mL of deionized water for 150 min, and the variation of the concentration of leached lead over time was monitored by inductively coupled plasma optical emission spectroscopy (ICP‐OES), as shown in Figure 5b. After 150 min of ageing, the concentration of Pb^2^⁺ ions in the pristine PSCs was 9.36 ppm, while the PSCs with CDVFB had a much lower value of 1.45 ppm. This indicates that CDVFB protection can effectively reduce lead leakage by more than 85%, which demonstrates that it is effective in protecting the integrity of PSCs.^[^ ^51^ ^]^ A strong interaction between CDVFB and perovskite is essential to stabilize lead ions and increase resistance to water, thus maintaining the structural integrity of the film and reducing the contamination of the environment.

The impact of CDVFB on device stability was subsequently evaluated through accelerated ageing in a maximum power point tracking (MPPT) model under continuous simulated illumination (100 mW cm^−2^) at a temperature range of 50–55 °C in ambient air. The initial efficiencies of the devices undergoing maximum power point tracking testing were 23.42% and 25.96%, respectively (Figure S34, Supporting Information). As depicted in Figure 5c, the evolution of the stabilization of unencapsulated devices occurs in two stages. During the first 100 h, the pristine device showed a rapid decline in efficiency, dropping to less than 80% of its initial value. This phenomenon is attributed to a number of water‐induced bulk chemical reactions associated with poor crystallization and low resistance to water. The performance of the pristine device then gradually declined until it reached ≈60% of its original efficiency after 1500 h. The PCE of PSCs treated with CDVFB showed only a decrease of 3%, which was 97% of the original efficiency. In addition, a damp‐heat stability assessment was conducted at 85 °C and 85% relative humidity for the encapsulated devices with and without CDVFB, using poly(3‐hexylthiophene) as the hole transport layer.^[^ ^52^ ^]^ The initial efficiencies of the encapsulated devices undergoing damp‐heat stability testing were 22.48% and 24.31%, respectively (Figure S35, Supporting Information). Over a period of 800 h, the devices without CDVFB experienced a significant decline in PCE to 44% of the initial value. Conversely, the CDVFB‐treated devices showed enhanced performance, and their PCE remained at 90% of the initial value. In order to provide further confirmation of the intrinsic moisture resistance that is conferred by CDVFB, damp‐heat stability tests were also carried out on unencapsulated devices under the same conditions (85 °C, 85% relative humidity, in the dark). The devices with CDVFB retained over 88% of their initial efficiency after 300 h, while the unencapsulated pristine devices decreased to 41% within 120 h (Figure S36, Supporting Information). The enhanced moisture and thermal stability of the devices with CDVFB can be ascribed to the improved water resistance of the highly branched fluorine‐containing polymer.

## 3 Conclusion

In summary, a branched fluorine‐containing polymer has been successfully developed by polymerizing a versatile organic monomer of DVFB to modulate the crystallization process of FA‐based perovskite film. The DVFB effectively inhibits complex intermediate phases, resulting in highly crystalline, low‐defect perovskite films with excellent phase purity and orderly crystal orientations. This approach results in a significant enhancement in solar cell performance, with a PCE of 26.05% for the small lab device (0.04 cm^2^) and a promising efficiency of 22.43% for the module device (16.1 cm^2^). The hydrophobic nature of CDVFB enhances the environmental stability of PSCs, as demonstrated by the retention of over 97% of their initial efficiency after 1500 h of continuous illumination (one sun intensity) under MPPT for the unencapsulated devices. These results demonstrate the efficacy of the CDVFB in overcoming the crystallization challenges inherent in FA‐based perovskite films processed by solution and at the same time improving the long‐term stability of the PSCs. The findings of this study provide a compelling path to the commercialization of stable, high‐efficiency perovskite solar cells and place DVFB as a key additive in the PV applications of the future.

## 4 Experimental Section

The details of film characterizations, device fabrication, and performance measurements are described in Supporting Information.

## Conflict of Interest

The authors declare no conflict of interest.

## Author Contributions

J.H. and X.L. contributed equally to this work. J.H. and M.W. conceptualized the project. J.H. was responsible for device fabrication. Film fabrication and characterization were performed by J.H., X.L., H.Y., X.M., W.Y., and L.D. DFT calculations were carried out by Z.Z. and T.S. GIWAXS measurements were conducted by H.D. Laser scribing was performed by L.W. and B.H. The original draft was written by J.H. and M.W., writing—review and editing—were done by J.H., S.Y., M.K.N., and M.W., and the project was supervised by S.Y., M.K.N., and M.W.

## Supporting information



Supporting Information

## Data Availability

The data that support the findings of this study are available in the supplementary material of this article.
