# Supplementary material for: Oriented Crystallization of Perovskite Film via Fluorine‐Containing Hyperbranched Polymer for Efficient and Stable Perovskite Solar Cells
Source: Adv Mater. 2025 Sep 18;38(2):e11684. doi: 10.1002/adma.202511684 (PMC12783880; doi:10.1002/adma.202511684)
Supplement: Supplementary file 1 — Supporting Information [file ADMA-38-e11684-s001.docx]

Supporting Information

Oriented Crystallization of Perovskite Film via Fluorine-containing Hyperbranched Polymer for Efficient and Stable Perovskite Solar Cells

Junyi Huang, Xiongjie Li, Zhiguo Zhang, Tianyu Sun, Hongliang Dong, Haixuan Yu, Xiaoting Ma, Wanpeng Yang, Letian Dai, Lei Wang, Bing Hu, Yan Shen*, Mohammad Khaja Nazeeruddin*, and Mingkui Wang*

J. Huang, Dr. X. Li, Dr. Z. Zhang, T. Sun, H. Yu, X. Ma, W. Yang, Dr. L. Dai, Prof. B. Hu, Prof. Y. Shen, Prof. M. Wang

Wuhan National Laboratory for Optoelectronics, Huazhong University of Science and Technology, 1037 Luoyu Road, Wuhan 430074, Hubei, People's Republic of China

E-mail: [ciac_sheny@mail.hust.edu.cn](mailto:ciac_sheny@mail.hust.edu.cn), mingkui.wang@mail.hust.edu.cn

Dr. H. Dong

Center for High-Pressure Science and Technology Advanced Research, Pudong, Shanghai 201203, People's Republic of China

L. Wang, Prof. B. Hu

Wuhan Hero Optoelectronics Technology Co., LTD, 6 Huanglongshan North Road, East Lake High-Tech Development Zone, Wuhan, Hubei Province, People's Republic of China

Prof. M. K. Nazeeruddin

Institut des Sciences et Ingénierie Chimiques, Ecole Polytechnique Fédérale de Lausanne, Lausanne, 1015, Switzerland

E-mail: [mdkhaja.nazeeruddin@epfl.ch](mailto:mdkhaja.nazeeruddin@epfl.ch)

Prof. M. K. Nazeeruddin

Mechanical and Energy Engineering Department, College of Engineering, Imam Abdulrahman Bin Faisal University; Dammam, 34212, Saudi Arabia

Prof. M. Wang

Optics Valley Laboratory, Wuhan, Hubei 430074, People's Republic of China

**Experimental Section**

1. Material synthesis

*Material*: The etched indium tin oxide (ITO) glass sheets (RS ≈8 Ω/sq), Formamidinium iodide (FAI, 99.9%), Nickel oxide (NiO) nanoparticles, [6,6]-phenyl-C61-butyric acid methyl ester (PC61BM) and 2, 2′′, 7, 7′′-Tetrakis [N, N-di(4-methoxyphenyl) amino]-9, 9′′-Spirobifluorene (Spiro-OMeTAD, 99.9%) were purchased from Advanced Election Technology CO., Ltd (Yingkou, China). [2-(3,6 dimethoxy-9h-carbazol-9-yl)ethyl]phosphonic acid (MeO-2PACz, >99%), bathocuproine (BCP, >99%) and Lead Iodide (PbI_2_, >99%) were acquired from TCI (Japan). Butylammonium Iodide (BAI, 99.5%), Methylammonium Iodide (MAI, 99.5%), methylammonium chloride (MACl, 99.9%), Cesium chloride (CsCl, 99.9%), Cesium Iodide (CsI, 99.9%), 4-test-butyl pyridine (4-tBP, 96%) and bis (trifluoromethyl sulfonyl)-imide lithium salt (Li-TFSI) were purchased from Xi’an Yuri Solar Co., Ltd. SnO_2_ (Tin (IV) oxide, 15% in H_2_O) colloidal dispersion liquid was purchased from Alfa Aesar. Dimethylsulfoxide (DMSO, 99.9%), N, N-dimethylformamide (DMF, 99.9%), chlorobenzene (CB, 99.9%), and isopropanol (IPA, 99.9%) were purchased from Sigma–Aldrich. I_2_ was purchased from Macklin.

*Synthesis of the* *1,4-divinyloctafluorobutane (DVFB)*: In a 1 L reactor, 35 g (0.138 mol) of I_2_ was added. After three cycles of high-purity nitrogen purging, 31.74 g (0.317 mol) of tetrafluoroethylene was introduced, and the mixture was heated to 150 °C with stirring. The reaction was stopped after 2 hours, and the reactor was cooled to room temperature to release unreacted tetrafluoroethylene. Subsequently, 0.17 g (0.9 mmol) of CuI was added. After three cycles of purging and replacement, 19.32 g (0.69 mol) of ethylene was added, and the mixture was heated to 200 °C for 6 hours. Once the reaction was complete, the reactor was cooled to room temperature. After another three cycles of purging and replacement, 19.32 g (0.69 mol) of ethylene was added, and the reaction was carried out at 200 °C for another 6 hours. At the end of the reaction, 9.27 g (0.166 mol) of KOH and 100 mL of ethanol were introduced into the reactor. The reaction mixture was refluxed at 90 °C with stirring for 20 minutes and then cooled to room temperature. The final product was obtained as 32 g with a yield of 95%.^[1]^

*Preparation of Solutions*: The SnO_2_-KCl solution was prepared by mixing 15% SnO_2_ hydrocolloid solution with 10 mg⋅mL^-1^ KCl solution at a volume ratio of 1:1. The mixed solution was stirred for 2 h, then filtered by a 0.22 µm syringe filter before use. A precursor solution of Cs_0.05_MA_0.05_FA_0.9_PbI_3_ was prepared by weighing PbI_2_ 645.4 mg, FAI 216.7 mg, MAI 11.1 mg, CsCl 11.8 mg, and MACl 24.5mg and then dissolving them in a 1mL mixed solvent of DMSO and DMF (volume ratio 1:4). For the DVFB precursor solution system, the concentration of DVFB in the perovskite precursor was 0.5-2 mg mL^–1^. For the hole transport layer, 72.3 mg Spiro-OMeTAD was dissolved in 1 mL chlorobenzene, stirred for 20 min, then 17.5 μL Li-TFSI (520 mg Li-TFSI dissolved in 1 mL acetonitrile) and 28.5 μL tBP was added. The Spiro-OMeTAD solution was obtained by stirring at room temperature for 12 h. The NiOx nanoparticle ink was prepared by 10 mg NiOx nanoparticle in a 1mL mixed solvent of H_2_O and IPA (volume ratio 3:1).

2. Device fabrication

*Small-sized n-i-p Solar Cell Fabrication*: The ITO was cleaned by ultrasonication in a cleaning liquid, deionized water, acetone, and anhydrous ethanol for 30 min, respectively, and then dried with nitrogen flow before use. The ITO glass substrates were treated with UV ozone for 30 min before depositing the SnO_2_ layer. The prepared SnO_2_-KCL solution was spin-coated onto the ITO substrate at 4000 rpm for 20 s followed by annealing at 150 °C for 20 min. For modified ITO glass/SnO_2_, 4-(Heptafluoropropan)-2-methylaniline (C3F7-MA) solutions of 0.02 mol⋅L^-1^ were prepared by dissolving them into IPA, and then the C3F7-MA solution was spin-coated on the ITO glass/SnO_2_ substrate at 5000 rpm for 20 s and annealed at 100 ℃ for 10 min. After cooling down to room temperature, the modified ITO glass/SnO_2_ substrates were transferred into the glove box. The prepared Cs_0.05_MA_0.05_FA_0.9_PbI_3_ perovskite precursor solution and the DVFB-based Cs_0.05_MA_0.05_FA_0.9_PbI_3_ perovskite precursor solution was spin-coated on the modified ITO glass/SnO_2_ substrate at 1000 rpm for 5s and 4000 rpm for 30s where 200 µL of CB anti-solvent was dripped on the perovskite films at 15 s before ending the program. The samples were then annealed at 100 ℃ for 60 min and 150℃ for 10 min. After the sample acquired room temperature, we spin-coated the BAI (5mg mL^−1^, IPA solution) at a speed of 6000 r for 20 s and annealed at 100 °C for 10 min. To deposit the hole transport layer (HTL), we spin-coated the prepared Spiro-OMeTAD precursor onto the perovskite film surface (5000 rpm, 20 s). Subsequently, we deposited a gold electrode, approximately 100 nm in thickness, onto the HTL using vacuum thermal evaporation.

*Small-sized p-i-n Solar Cell Fabrication*: The ITO was cleaned by ultrasonication in a cleaning liquid, deionized water, acetone, and anhydrous ethanol for 30 min, respectively, and then dried with nitrogen flow before use. The ITO glass substrates were treated with UV ozone for 30 min before depositing the NiOx layer. Then, the prepared NiOx nanoparticle ink was spin-coated onto the ITO substrate at 3000 rpm for 20 s, then annealed at 100 °C for 10 min under air environment and then transferred to a N_2_-filled glovebox. Subsequently, the solution of Me-2PACz with the concentration (0.5 mg L^−1^ in ethanol) was deposited on the ITO/ NiOx substrate at 3000 rpm for 30 s, annealing at 100 °C for 10 min to yield ITO/NiOx/Me-2PACz HTL. The prepared Cs_0.05_MA_0.05_FA_0.9_PbI_3_ perovskite precursor solution and the DVFB-based Cs_0.05_MA_0.05_FA_0.9_PbI_3_ perovskite precursor solution was spin-coated on the modified ITO/NiOx/Me-2PACz substrate at 1000 rpm for 5s and 4000 rpm for 30s where 200 µL of CB anti-solvent was dripped on the perovskite films at 15 s before ending the program. The samples were then annealed at 100 ℃ for 60 min and 150℃ for 10 min. After the sample acquired room temperature, we spin-coated the PEACl (1mg mL^−1^, IPA solution) at a speed of 4000 r for 20 s and annealed at 100 °C for 10 min. Next, PCBM solution (20 mg mL^−1^ in CB) was spin-coated on the perovskite surface at 2500 rpm for 20 s. BCP solution (saturated in IPA) was spin-coated at 5000 rpm for 20 s. Subsequently, we deposited a silver electrode, approximately 100 nm in thickness, onto the BCP using vacuum thermal evaporation.

*Module Fabrication*: Perovskite solar minimodules, with 7 sub-cells connected in series, were fabricated on ITO glass substrates with a size of 5×5 cm^2^. The series interconnection of the module was realized by P1, P2, and P3 lines, which were patterned using a laser scribing system with a 1064 nm and a power of 20 W. The ITO substrate was pre-patterned for P1 (width of 80 μm) using 30% laser power under a speed of 300 mm s^–1^ with a frequency of 65 kHz. The subsequent processes for the preparation before the Au layer are the same as the small-area device procedures. The P2 lines (a width of 600 μm) were patterned before the Au evaporation process step with an average laser power of 15% under a speed of 300 mm s^–1^ and frequency of 65kHz. The distance between P1 and P2 was about 60 μm. Subsequently, we deposited a gold electrode, approximately 100 nm in thickness, onto the HTL using vacuum thermal evaporation. The P3 (a width of 100 μm) is fabricated under the same scribing condition as the P2 line. The distance between P2 and P3 was also about 60 μm.

3. Material characterization and measurements

*Characterization and Measurements*: We utilized a xenon light source solar simulator (450W, Oriel, model 9119) equipped with an AM 1.5G filter (Oriel, model 91192) to deliver an irradiance of 100 mW cm^–2^ at the surface of the solar cells. The photocurrent-voltage (*J–V*) characteristics of the 0.04 cm^2^ and 16.1 cm^2^ size devices were measured through a shadow mask under these conditions by applying an external potential bias to the devices and measuring the resulting photocurrent using a Keithley model 2400 digital source meter. The IPCE values for bias light-free devices were measured in the wavelength range of 300 to 900 nm using IQE200B, Oriel. The surface morphology was examined by using field-emission scanning electron microscopy (FSEM, Nova NanoSEM 450). Elemental distributions were determined using transmission electron microscope (TEM) operating at an energy resolution of 136 eV. For ToF-SIMS analysis, a 25 keV pulsed Bi^+^ primary ion beam for analytical puposes was employed. The examined area was 100 × 100 μm^2^. X-ray diffraction (XRD) pattern was recorded by utilizing an XRD diffractometer with Cu Kα radiation from GKINST Co., LTD. In-situ XRD experiment was performed on a Smart Lab 9KW diffractometer equipped with a planar detect with a high temperature stage, which allows sample to be measured at controlled temperatures. Grazing incidence wide-angle x-ray scattering (GIWAXS) measurements were recorded at the Shanghai Synchrotron Radiation Facility. For synchrotron GIWAXS measurements, all the samples were prepared with perovskite solutions on quartz substrates. Fourier transforms infrared spectra (FTIR) spectra were obtained using an FTIR spectrometer equipped with a diamond ATR (Nicolet iS50R, Thermo Scientific). ^1^H NMR spectra were measured on a Bruker 600 MHz spectrometer in DMSO-d_6_. The temperature was kept constant at 294 ± 0.2 K during measurements. Ultraviolet–visible absorption light absorption spectra were recorded using a PE950 spectrophotometer. The in situ ultraviolet–visible absorption light absorption spectra were acquired on a set-up equipped with an in situ spectra monitoring system (Du100, PuGuangWeishi). A continuous halogen-lamp light source was utilized for the ultraviolet–visible spectra measurements. Photoluminescence (PL) measurements were conducted employing a fluorescence spectrophotometer (LabRAM HR800) with excitation at 532 nm. Time-resolved PL (TRPL) decay kinetics were determined using a 478 nm light pulse as excitation, employing the Delta Flex Fluorescence Lifetime System (Horiba Scientific Com., Japan). Fluorescence lifetime imaging microscopy was conducted using FLIM300 (Dalian Chuangrui Spectral Technology Co., Ltd). The samples were uniformly deposited on glass substrates following the identical procedure used for device fabrication. Measurements involving X-ray photoelectron spectroscopy (XPS) were conducted using a photoelectron spectrometer (AXIS-ULTRA DLD-600W, Kratos, Shimadzu, Japan). Photovoltage/photocurrent transient decay measurements were carried out to obtain the recombination/transport lifetime of solar cell devices (Hua Ming, model III). A white-light bias on the device sample was generated from an array of diodes. A pulsed green laser was used as the perturbation source, with a pulse width of 10 ns and a repetition frequency of 10 Hz. Transients were measured at different white light intensities via tuning the voltage applied to the bias diodes. The voltage/current output was recorded on an oscilloscope directly connected with the cells.

*DFT calculations:* The first principles computations were carried out by Vienna Ab initio Simulation Package (VASP) based on density functional theory. Generalized gradient approximation (GGA) with the Perdew-Burke-Ernzerhof (PBE) functional was used to describe the exchange-correlation interaction. The projector augmented wave pseudopotentials were adopted to describe the ion-electron with a cutoff energy of 500 eV. The K-point grid of Brillouin zone was tested and sampled by 3×3×1 within Gamma-Pack. The electronic energy and forces were converged to within 10^-5^ eV and 0.03 eVÅ^-1^, respectively. The electrostatic potentials (φ) of the molecule were calculated using the Gaussian 09 package at the B3LYP/def2TZVP level with DFT-D3. The surface binding energy for adsorbent A on substrate B is calculated as Eb=E_A/B_-E_A_-E_B_ where E_A/B_, E_A_ and E_B_ are the energies of the adsorbing system A/B.

**Figure S1.** Synthetic route of DVFB.


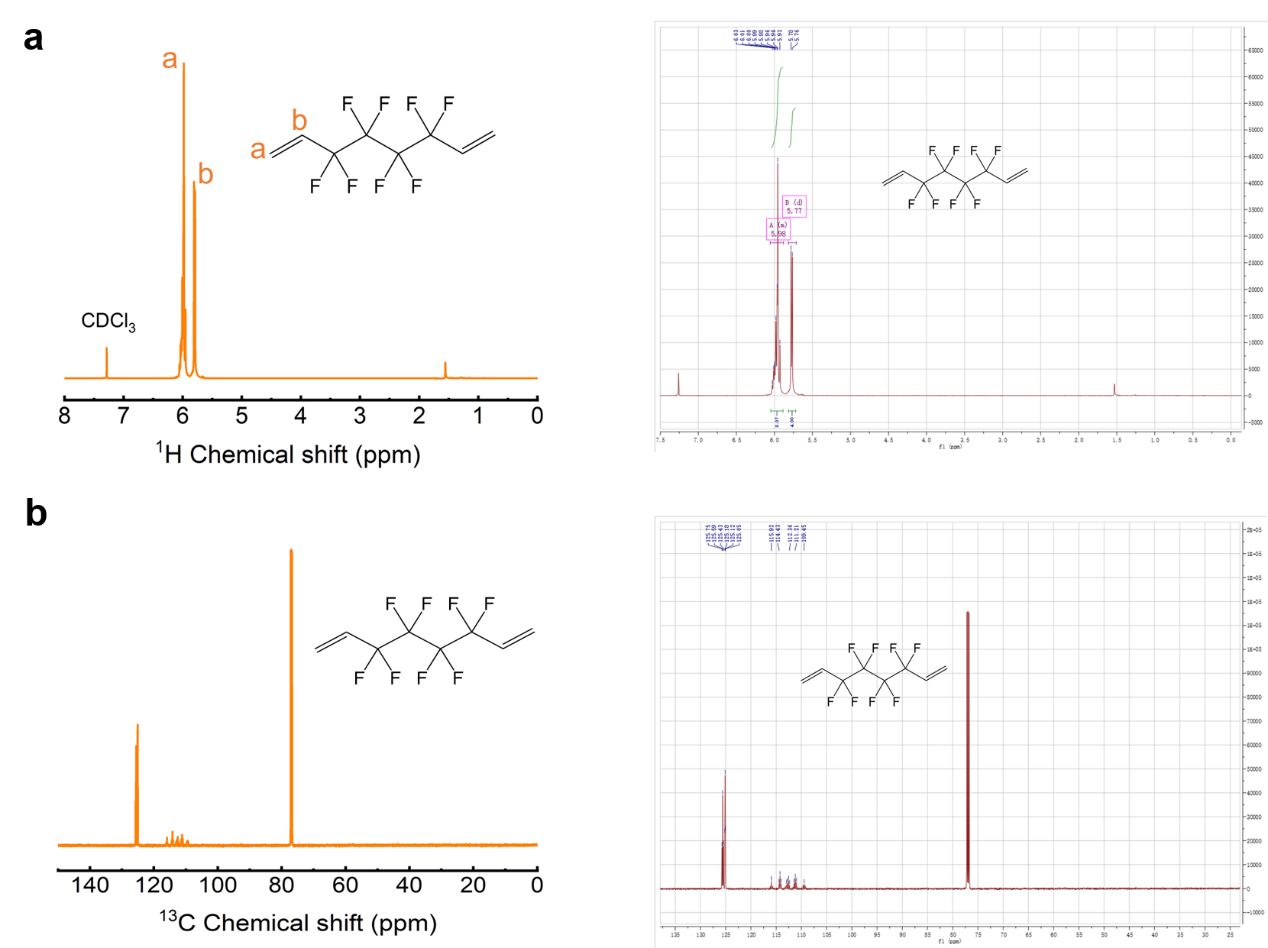


**Figure S2.** a) ^1^H NMR spectrum of DVFB, ^1^H NMR (600 MHz, CDCl_3_) δ = 6.20 – 5.89 (m, 4H), 5.89 – 5.68 (m, 2H). b) ^13^C NMR spectrum of DVFB, ^13^C NMR (CDCl_3_) δ = 125.32 (dt, *J*=18.8), 115.92 (t, *J*=29.7), 114.27 (dd, *J*=39.5), 113.44 - 112.06 (m), 111.11 (dd, *J*=67.3), 110.00 - 108.70 (m).

Figure S2 shows the ^1^H NMR and ^13^C NMR of DVFB. The ^1^H NMR spectrum shows two characteristic doublets at δ 6.20 – 5.89 ppm and δ 5.89 – 5.68 ppm, each of which integrates to two protons. These correspond to the vinyl groups (–CH=CH_2_) at both ends of the molecule. The ^13^C NMR spectrum displays multiple peaks, one of which, at δ 125.32 (dt, *J* = 18.8), corresponds to the vinyl carbons adjacent to the fluorinated chains. δ 115.92 (t, *J* = 29.7), 114.27 (dd, *J* = 39.5), and 111.11 (dd, *J* = 67.3) are assigned to the CF_2_-bearing carbons.

**Figure S3**. Mass spectrum of DVFB. The mass spectrum shows a dominant molecular ion peak at m/z = 254, which matches the calculated molecular weight of DVFB.


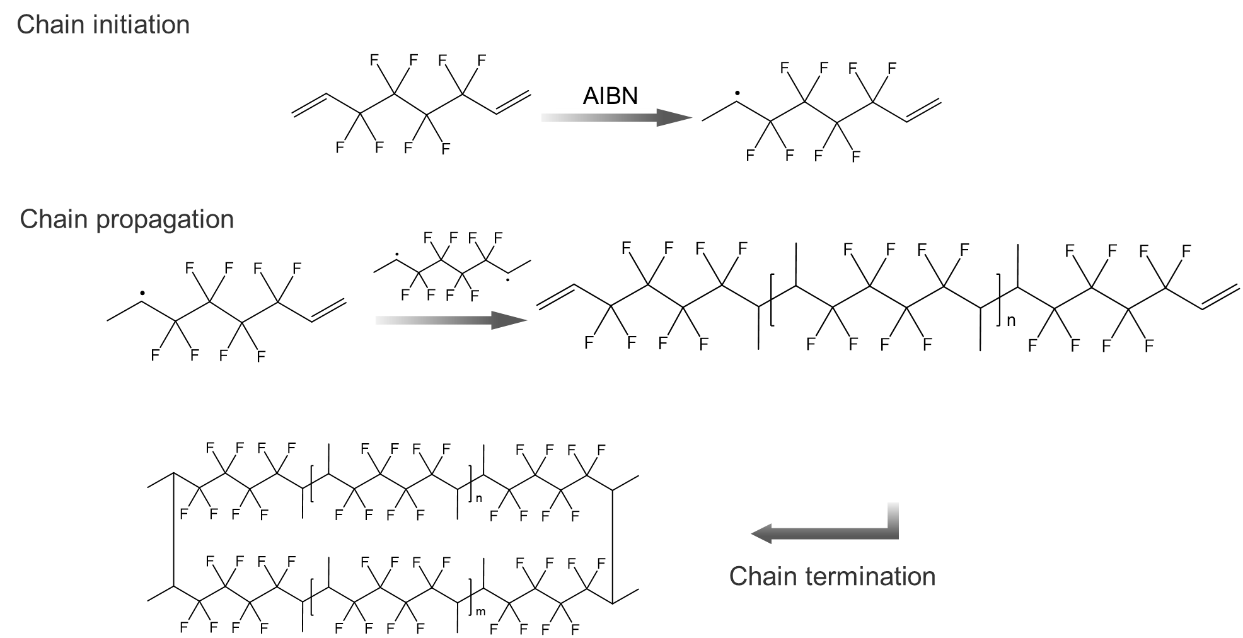


**Figure S4.** Detailed cross-linking process of DVFB.


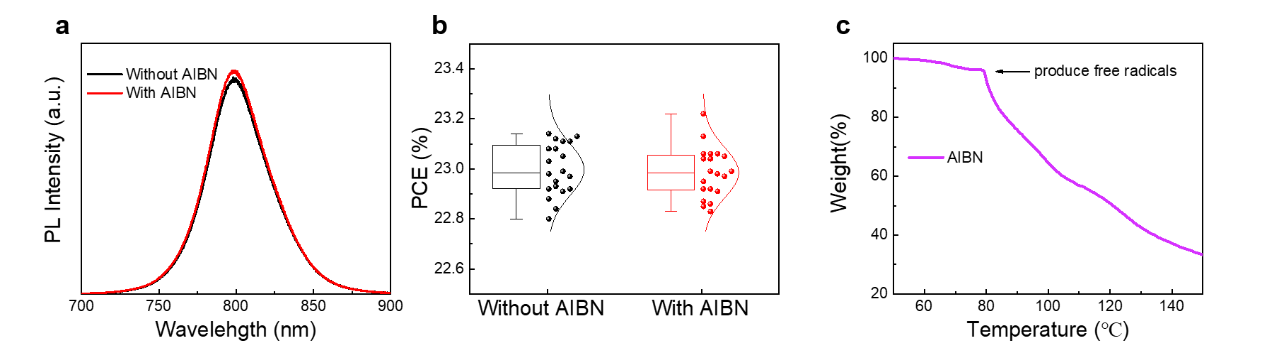


**Figure S5**. a) PL spectra of the perovskite with and without AIBN. b) Photovoltaic parameters statistics of PCE based on 20 devices with or without AIBN. c) Thermogravimetric analysis curves of AIBN.

AIBN was introduced as a free radical initiator in the perovskite precursor to initiate the cross-linking of DVFB via thermal radical polymerization during annealing. Specifically, the molar concentration of perovskite in the precursor is in the range of millimoles, whereas DVFB is added at a concentration of 1.5 mg mL^–1^, with AIBN accounting just 0.01 mol% of the DVFB amount. This means the AIBN content is approximately one ten-thousandth of the perovskite content on a molar basis. Such a trace amount of AIBN has a negligible influence on the optical performance and photovoltaic performance (Figure S5a, b). Furthermore, thermogravimetric analysis (TGA, Figure S5c) reveals that AIBN begins to decompose at approximately 80 °C, generating free radicals that initiate polymerization. It is completely decomposed at temperatures exceeding 100 °C. Since perovskite annealing is typically conducted at or above this temperature, the AIBN is entirely consumed during this step, leaving no residue in the final film.

**Figure S6**. The photographs of DVFB a) before and b) after heating.

**Figure S7.** The enlarged FTIR spectrum highlights the C=C stretching region for DVFB-containing PbI_2_ films before and after thermal annealing.


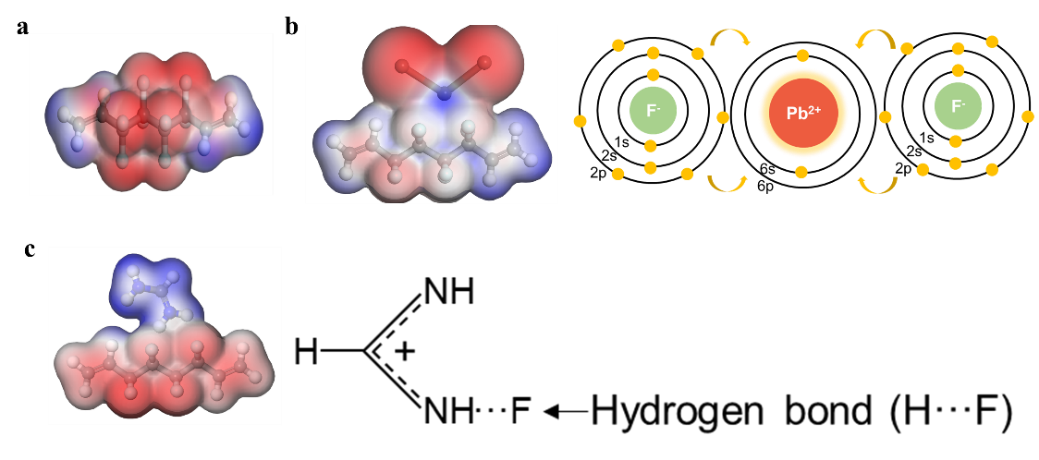


**Figure S8.** Calculated electrostatic potential mapping images for a) DVFB, b) DVFB+PbI_2_ and c) DVFB+FA^+^.

Note that the graphic has “blue and red” regions, corresponding to electron-poor and electron-rich regions, respectively. For DVFB molecules, there are many F atoms in the “red” regions act as Lewis bases, donating their lone pair electrons into the vacant 6p orbitals of Pb^2+^ ions, thereby forming directional Pb–F coordination bonds with a bond length of ~2.3 Å and a binding energy of −1.76 eV (Figure S5).^[2]^ This covalent interaction is evidenced by the downshifted UV-visible absorption (Figure 1d) and Pb 4f5/2 and 4f7/2 XPS peaks (Figure S6), indicative of electron transfer from F to Pb^2+^.

Simultaneously, the high electronegativity of F induces a pronounced polarization of the σ-bonding electron density in the N–H bonds of Formamidinium cations (FA⁺, CH(NH_2_)_2_⁺), causing a partial charge separation (δ⁺ on H, δ⁻ on F). This electrostatic interaction drives the formation of linear N–H···F hydrogen bonds (bond length: ~2.7 Å, ∠N–H···F >150°), where the lone pair electrons of F engage in a directional dipole-dipole interaction with the proton donor (N–H),^[3]^ as evidenced by the splitting of FA⁺ NH₂ proton signals in ^1^H NMR (8.75 ppm → 8.91/8.61 ppm, Figure 1b) and the red-shifted C–F stretching vibration in FTIR (1405 → 1398 cm⁻^1^, Figure 1c).


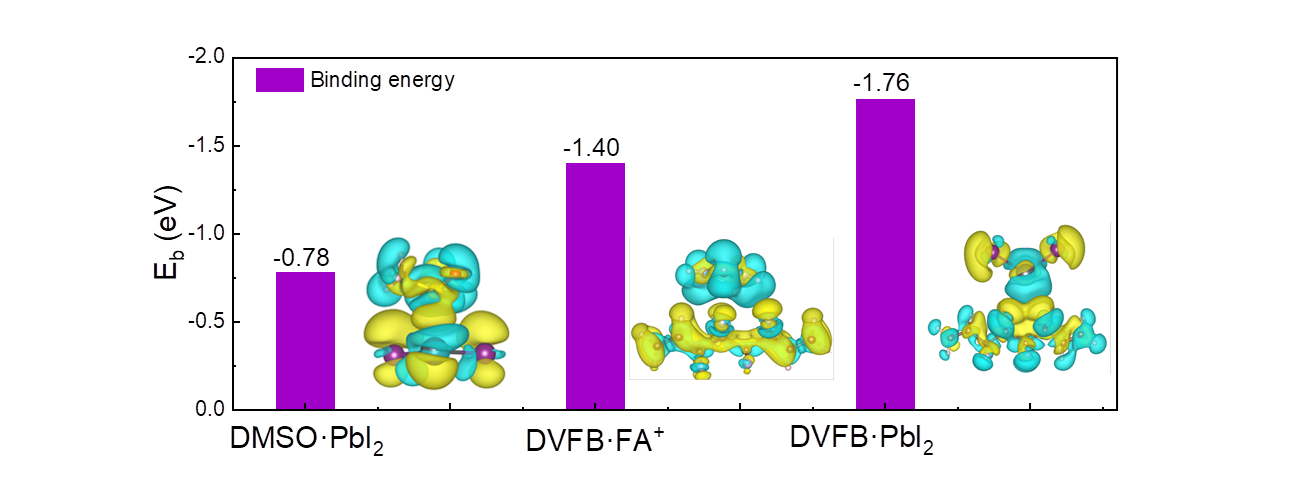


**Figure S9.** Binding energies between PbI_2_+DMOS, DVFB+FA^+^, DVFB+PbI_2_.

**Figure S10.** a) Pb 4f XPS spectra of the perovskite film with and without DVFB, and b) F 1s XPS spectra of the perovskite film with DVFB and pure DVFB film.

**Figure S11.** The time evolution of peak areas of the perovskite a) without and b) with DVFB, extracted from the XRD spectra.


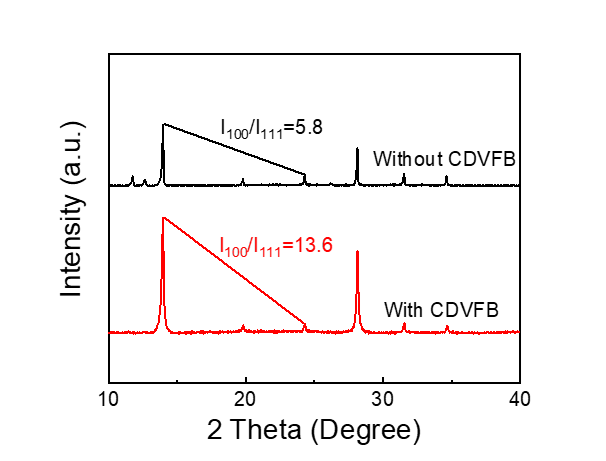


**Figure S12.** XRD patterns of the perovskite films after annealed at 100 ℃ for 1h and 150 ℃ for 10 min.


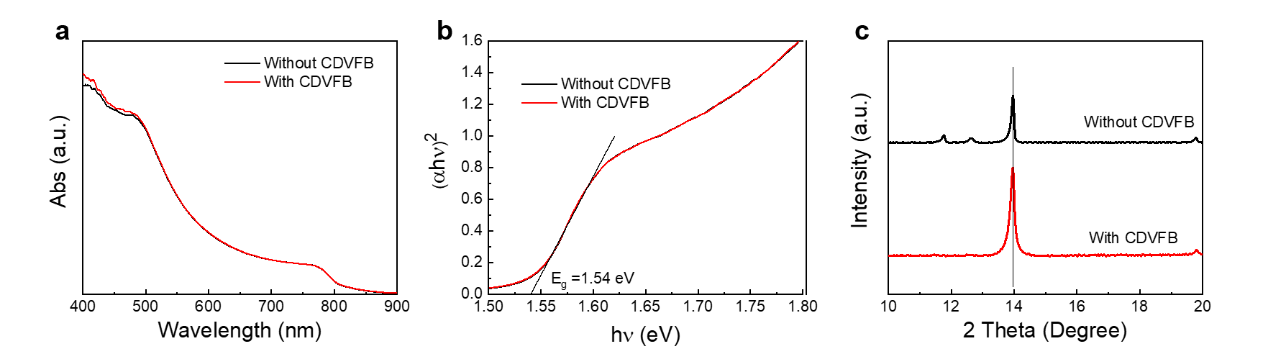


**Figure S13.** a) UV-vis absorption spectra and b) the extracted bandgap value of the perovskite film with and without CDVFB treatment. c) XRD patterns of the perovskite films with and without CDVFB treatment.


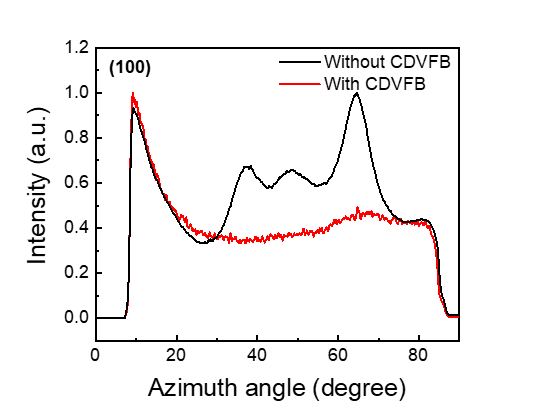


**Figure S14.** The azimuthal integration of (100) crystal plane of GIWAXS patterns.


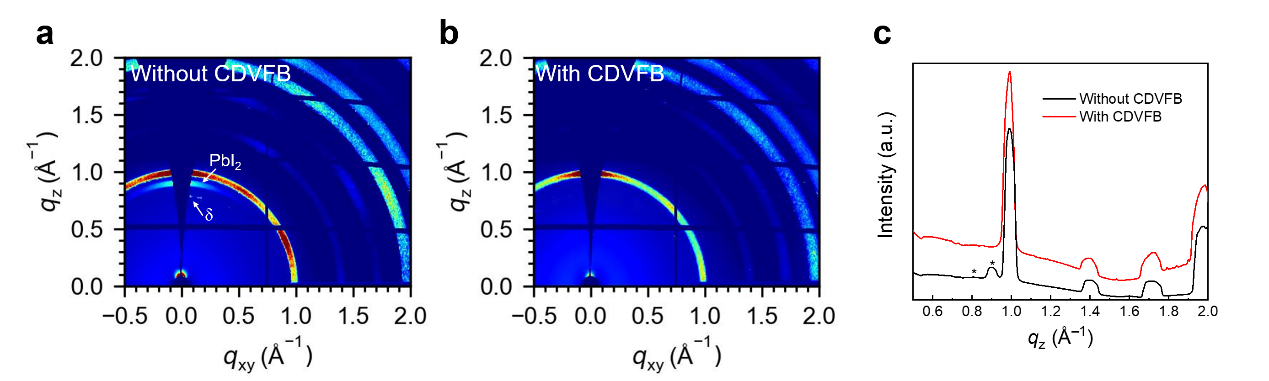


**Figure S15.** Expanded GIWAX patterns of a) the perovskite film without CDVFB and e) the perovskite film with CDVFB after annealed at 100 ℃ for 1 hour and at 150 ℃ for 10 min. c) Line cuts of the GIWAXS data.

To further validate the structural differences observed in XRD and address the limited detection range for the GIWAXS measurements of Figures 2d and 2e, we conducted expanded GIWAXS measurements by stitching together multiple adjacent regions. The resulting composite patterns and line-cut profiles are shown in Figure S15. For the perovskite film without CDVFB, GIWAXS data exhibited characteristic of disordered crystallographic orientation, along with the presence of additional diffraction signals corresponding to the δ-phase perovskite and residual PbI_2_. This finding was consistent with the results of XRD analysis. In contrast, the perovskite film with CDVFB exhibits a more vertically aligned crystal orientation, as evidenced by the concentrated (100) diffraction in the low azimuthal angle. Furthermore, the δ-phase and PbI_2_ related peaks are significantly suppressed, indicating improved crystallinity and phase purity.


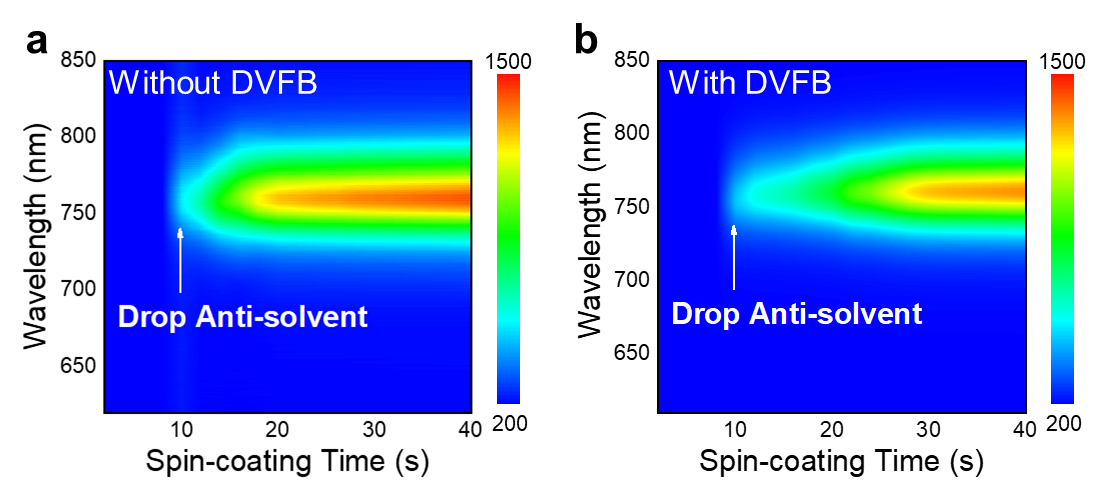


**Figure S16.** In-situ PL spectra of perovskite films fabricated a) without and b) with DVFB during the initial spin-coating process.


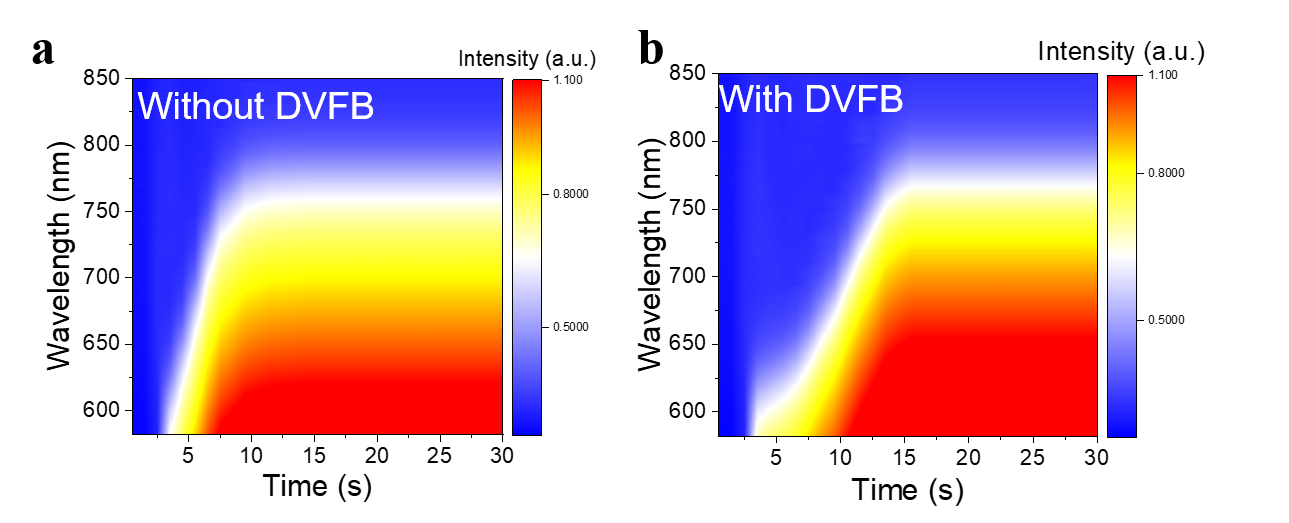


**Figure S17**. In-situ UV absorption spectra of perovskite films fabricated a) without and b) with DVFB during the initial annealing process.


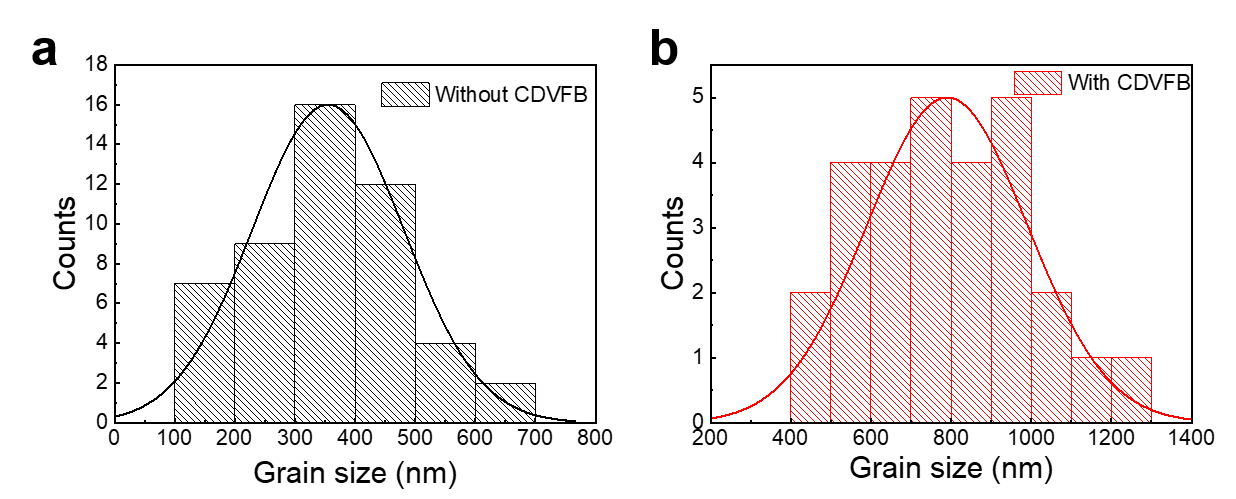


**Figure S18**. Distribution histogram of crystal grain sizes of the perovskite film a) without and b) with CDVFB, extracted from the SEM characterization.


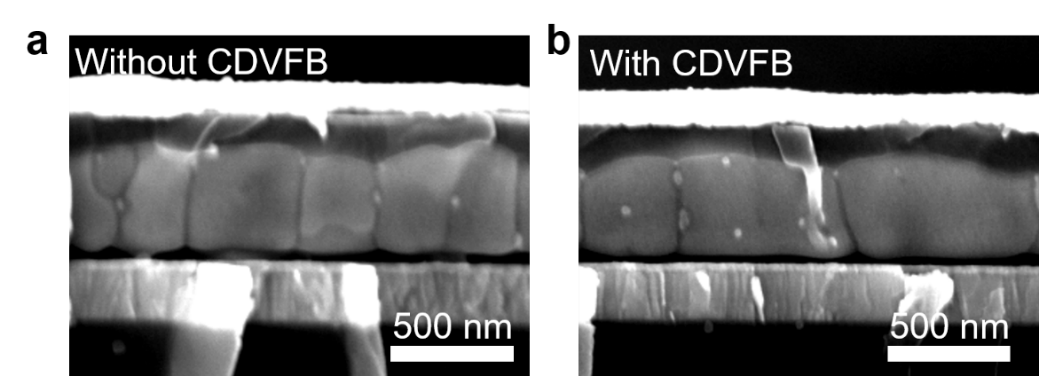


**Figure S19**. Cross-sectional SEM images of the perovskite devices a) without and b) with CDVFB.


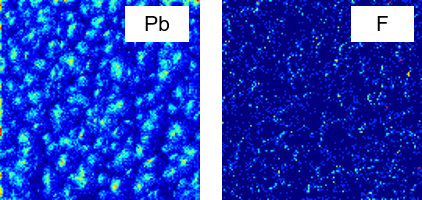


**Figure S20**. The ToF-SIMS 2D imaging of Pb and F distribution on the CDVFB-contained perovskite layer.


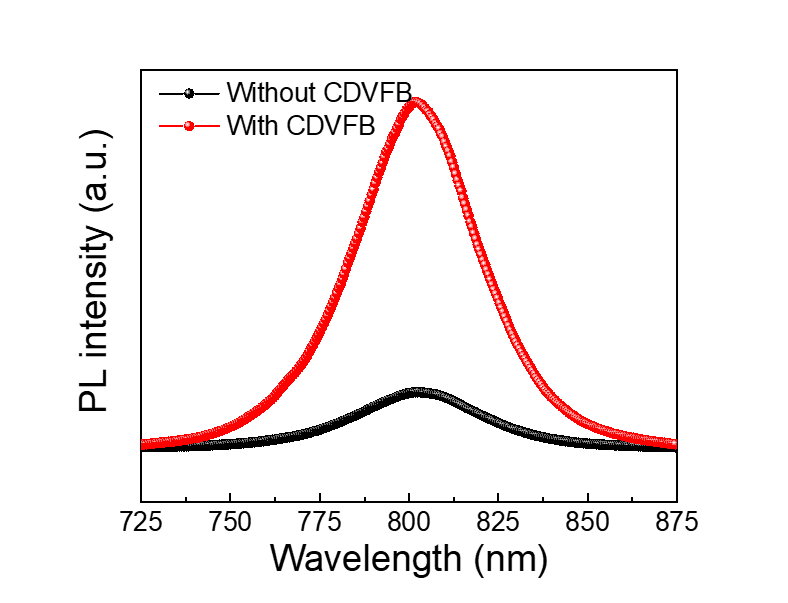


**Figure S21**. Comparison of the steady-state photoluminescence spectra.


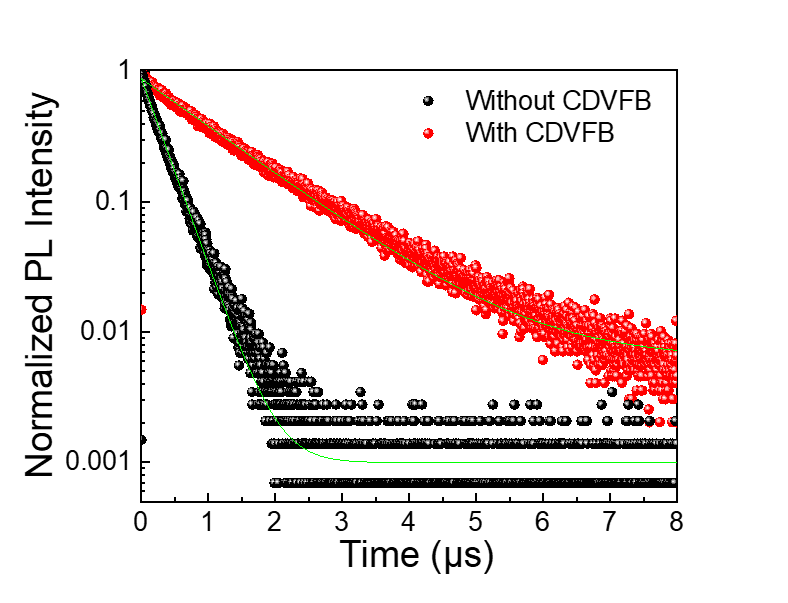


**Figure S22**. Comparison of the time-resolved photoluminescence spectra.


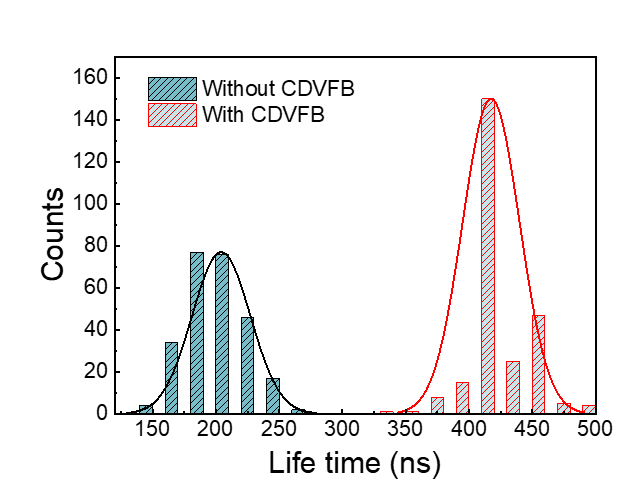


**Figure S23.** Distribution histogram of the lifetime of the perovskite film with and without CDVFB, extracted from the fluorescence lifetime imaging.


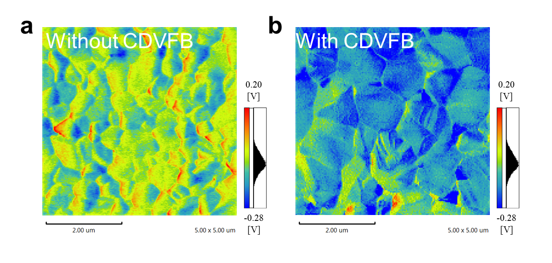


**Figure S24**. KPFM surface potential images of the perovskite films a) with and b) without CDVFB.

The KPFM images show that the potentials of the grain boundaries (GBs) of the perovskite films of the pure perovskite films are lower than those of the grain interiors (GIs). On the contrary, the potential of the GBs was significantly higher than that of the GIs for the perovskite films with CDVFB. This is due to the CDVFB located at the grain boundaries, which passivates the GBs and increases the contact potential difference at the grain boundaries.^[4,5]^ Additionally, the images reveal that CDVFB treatment leads to enlarged and more uniform grains, indicating improved crystallization. Although the average surface contact potential difference (CPD) of the film with CDVFB is slightly lower than that of the pristine film, this reflects a more p-type surface character. In inverted (p–i–n) devices, this shift may cause a minor loss in PCE, but the dominant effect of CDVFB-induced passivation at GBs and surfaces significantly improves carrier lifetime and suppresses non-radiative recombination, leading to improved overall device performance and long-term stability.


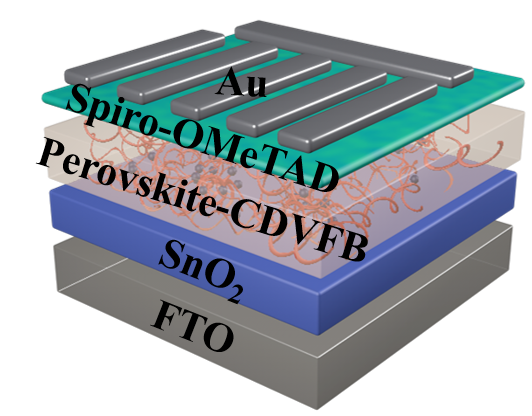


**Figure S25.** Schematic architecture of the PSC.


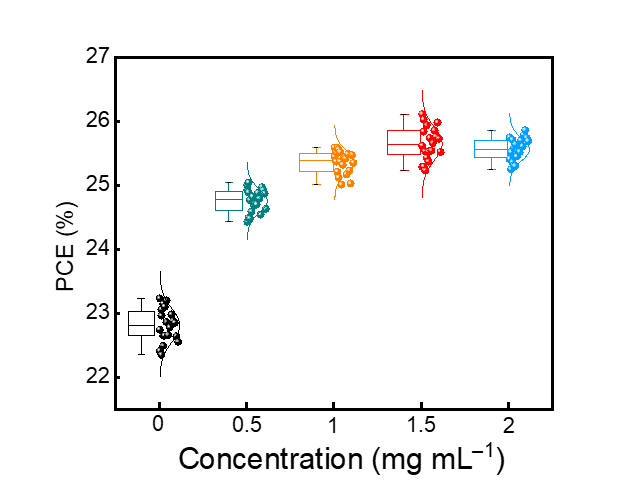


**Figure S26.** PCE of devices with different concentration of CDVFB.


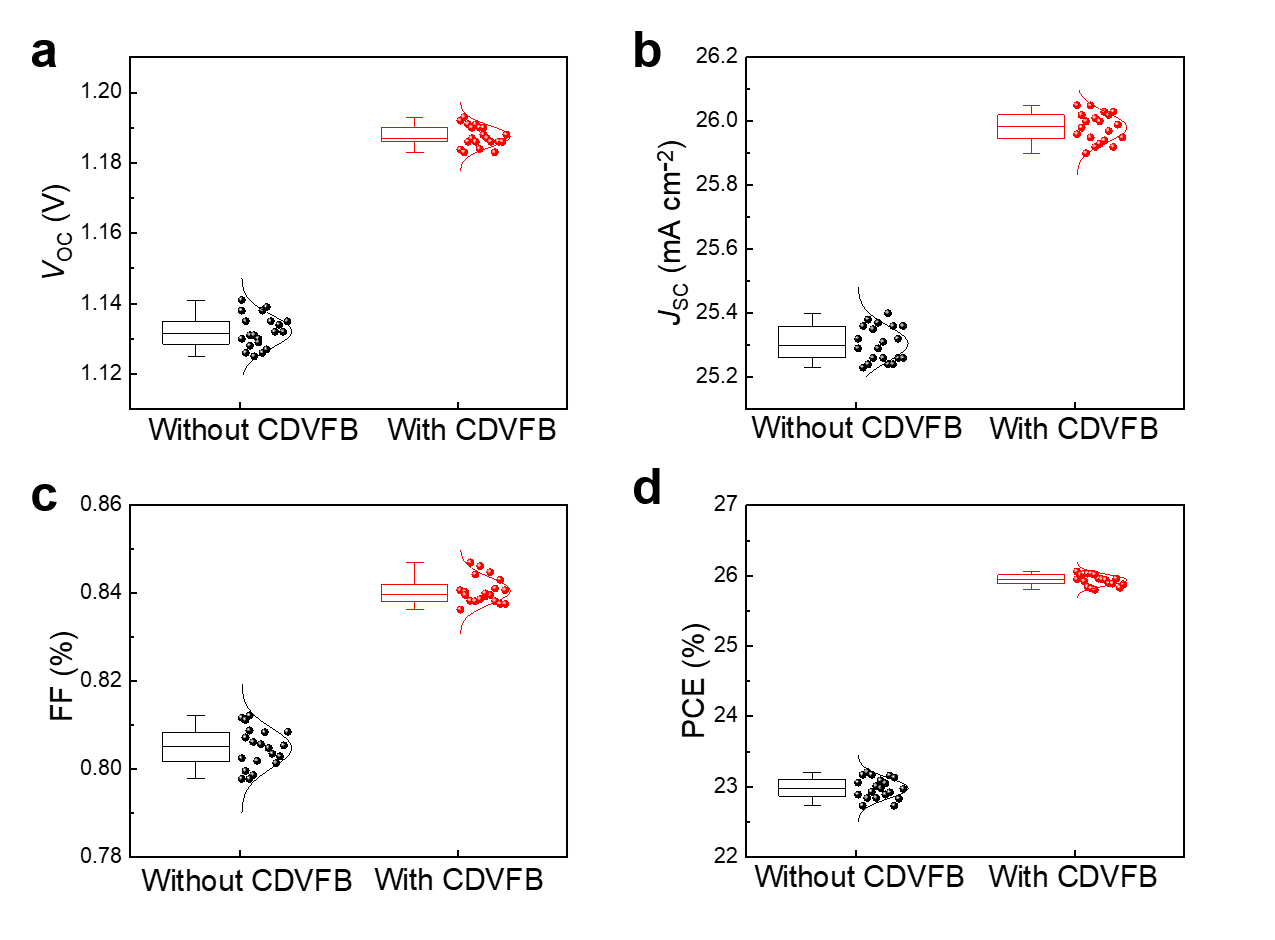


**Figure S27.** Photovoltaic parameters statistics of a) *V*_OC_, b) *J*_SC_, c) FF, and d) PCE based on 20 devices with or without CDVFB.


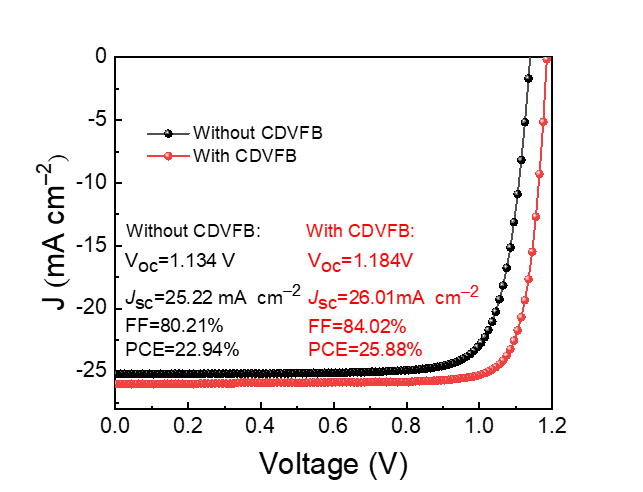


**Figure S28.** *J–V* curves of the best-performing regular (n-i-p) devices using perovskite treated with and without CDVFB measured under AM 1.5 G illumination of 100 mW cm^−2^ in the forward scan.

**
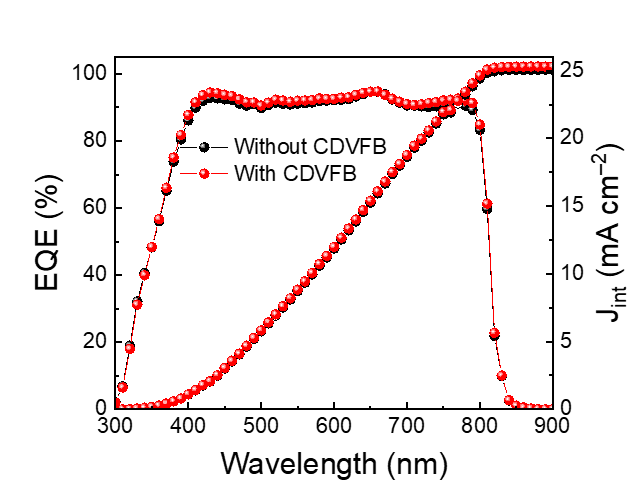
**

**Figure S29.** EQE spectra of the devices using perovskite with and without CDVFB.

**
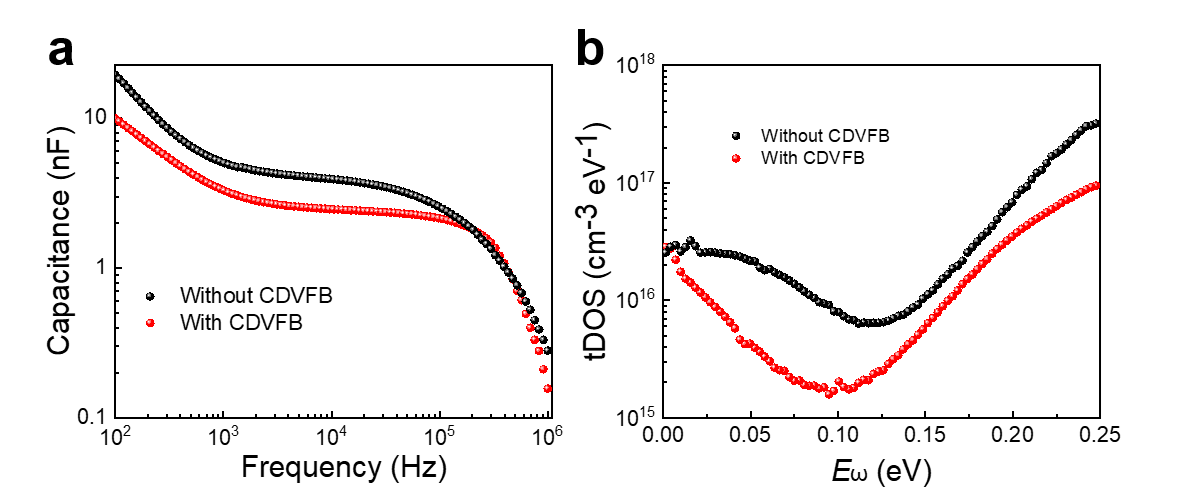
**

**Figure S30.** a) Capacitance vs. frequency measurement *via* PSCs with and without CDVFB. The measurement was performed at dark at room temperature. b) Defect density of states characterization.

Trap density curves for the control and target PSCs. To quantify the passivation effect of DVFB, Mott-Schottky analysis and trap density of states (*t*DOS) measurements were conducted on the devices.^[6]^ The trap density (*N*_T_) was determined by analyzing the angular-frequency-dependent capacitance. The target device exhibits reduced *t*DOS across all trap depths, as revealed by the angular-frequency-dependent capacitance according to the equation:

$$tDOS\left( E_{\omega} \right)=-\frac{V_{bi}}{qW}\frac{dC}{d\omega}\frac{\omega}{k_{B}T}$$

and

$$E_{\omega}=k_{B}Tln\left( \frac{\omega_{0}}{\omega} \right)$$

where *C* is the capacitance, ꞷ is the angular frequency, ꞷ_0_ is the attempt-to-escape frequency extracted from capacitance frequency spectroscopy, *q* is the elementary charge, *k*_B_ is the Boltzmann’s constant and *T* is the temperature (300 K in our measurement condition). *V*_bi_ and *W* are the built-in potential and depletion width.


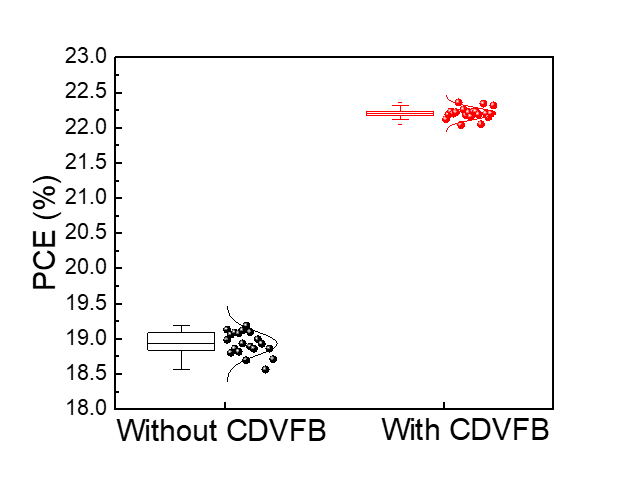


**Figure S31.** PCE parameters of perovskite modules with and without CDVFB.


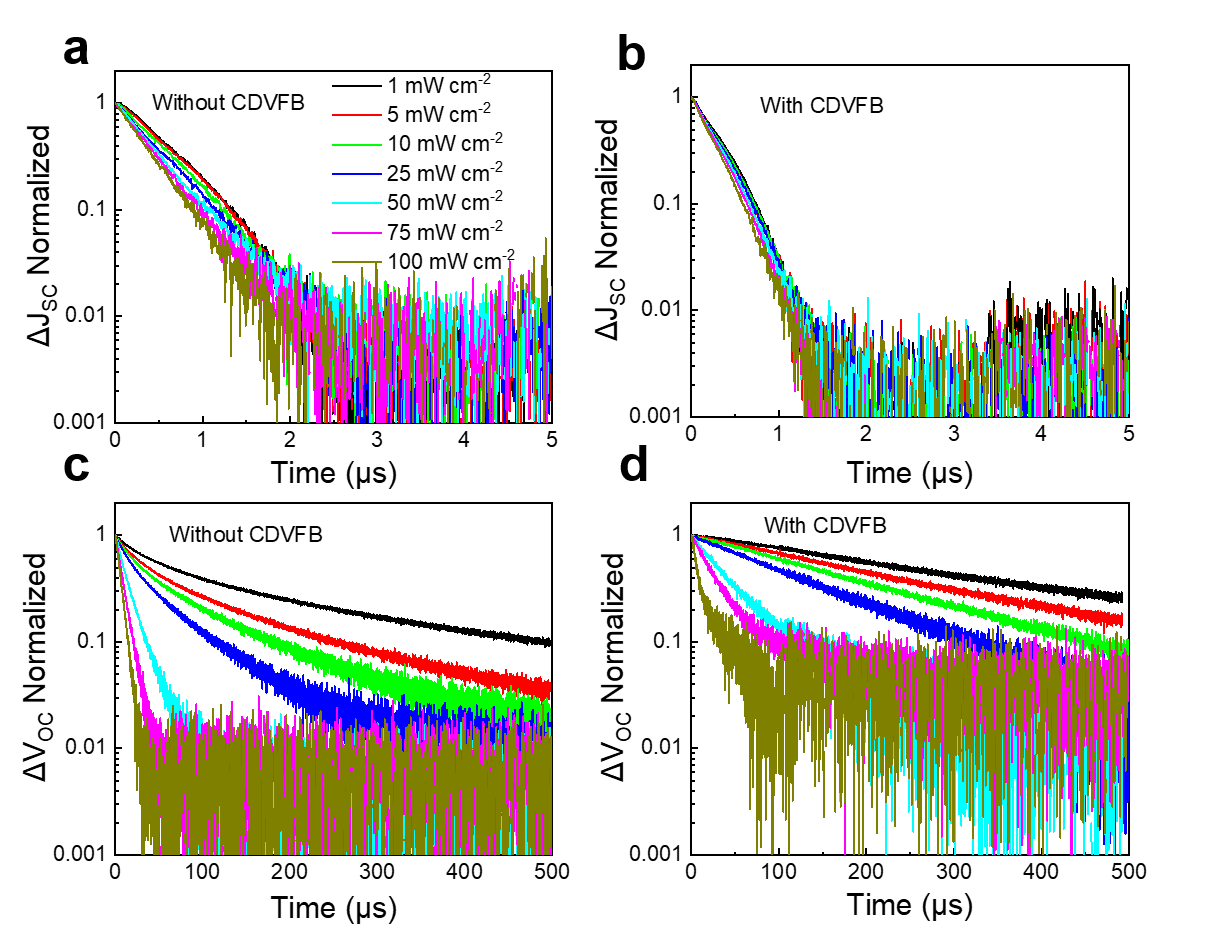


**Figure S32**. a-b) Transient photocurrent and c-d) Transient photovoltage decay curves of the device with and without CDVFB modified under varying light intensity.


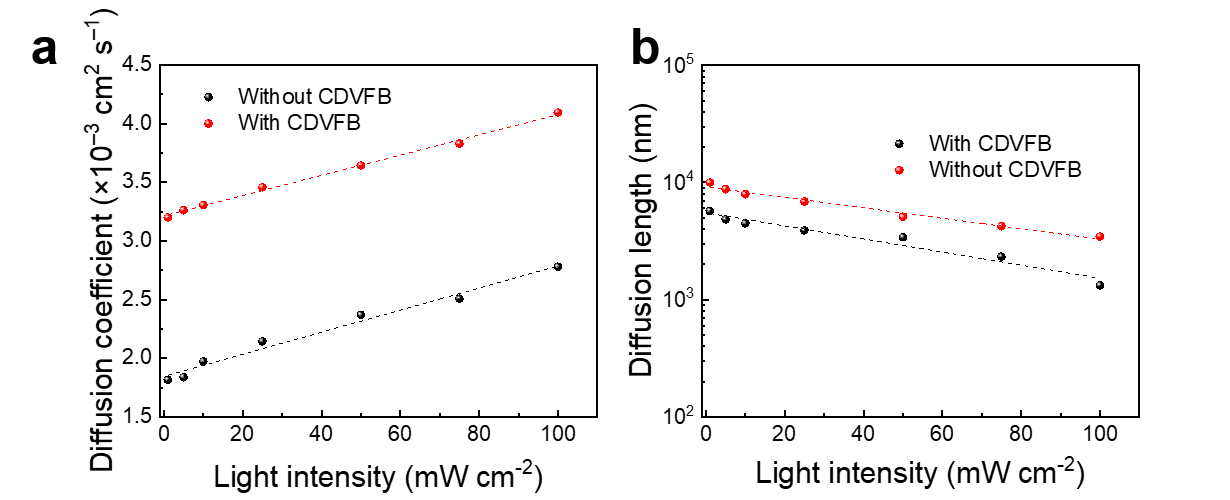


**Figure S33**. a) Carrier diffusion coefficients (D) and b) diffusion lengths (L) obtained from TPC/TPV decay measurements.


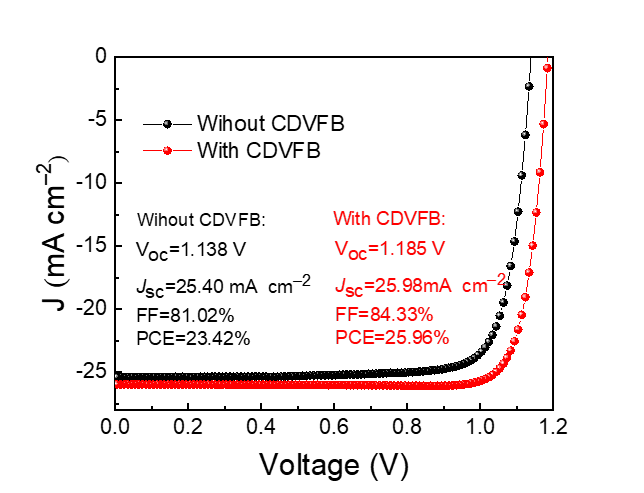


**Figure S34.** Initial *J–V* curve of the PSCs with and without CDVFB.


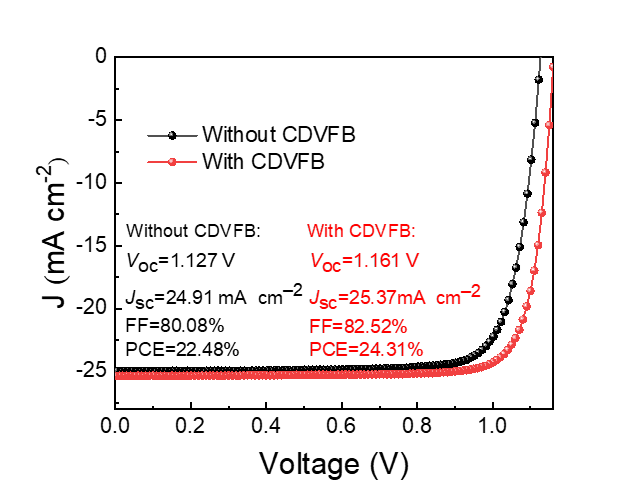


**Figure S35.** Initial *J–V* curve of the PSCs with and without CDVFB using P3HT as the hole transport layer.


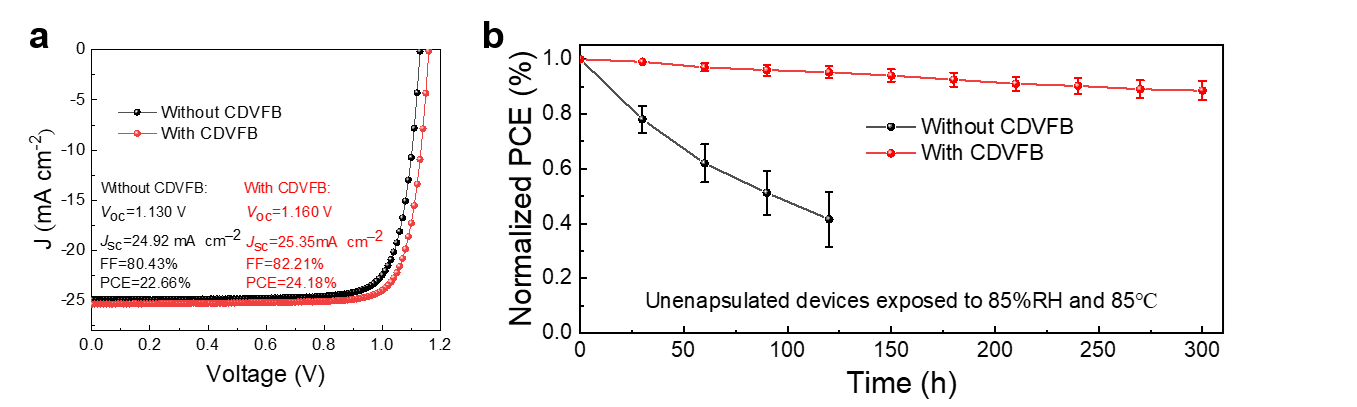


**Figure S36.** a) Initial *J–V* curve of the PSCs with and without CDVFB using P3HT as the hole transport layer. b) Stability measurements of unencapsulated devices aged under 85 °C and 85% RH in the dark.

**Table S1**. Photovoltaic parameters of the 16.1 cm^2^ sized perovskite module.

| Device | *V*_OC_  [V] | | *J*_SC_  [mA⋅cm^–2^] | FF  [%] | PCE  [%] |
| --- | --- | --- | --- | --- | --- |
| Without CDVFB | | 7.47 | 3.57 | 71.81 | 19.15 |
| With CDVFB | | 7.81 | 3.63 | 79.11 | 22.43 |

**References**

[1] K. Baum, C. D. Bedford, R. J. Hunad, *J. Org. Chem.* **1982**, *47*, 2251.

[2] R. Sun, Q. Tian, M. Li, H. Wang, J. Chang, W. Xu, Z. Li, Y. Pan, F. Wang, T. Qin, *Adv. Funct. Mater.* **2023**, *33*, 2210071.

[3] K. Dong, L. Zhu, G. Yang, L. Zheng, Y. Wang, B. Zhang, J. Zhou, J. Bian, F. Zhang, S. Yu, S. Liu, M. Wang, J. Xiao, X. Guo, X. Jiang, *ChemSusChem*, **2024**, *17*, e202400038.

[4] X. Yuan, R. Li, Z. Xiong, P. Li, G. O. Odunmbaku, K. Sun, Y. Deng and S. Chen, *Adv. Funct. Mater.*, **2023**, *33*, 2215096.

[5] Z. Wu, H. Cai, T. Wu, J. Xu, Z. Wang, H. Du, J. Zhao, F. Huang, Y.-B. Cheng, J. Zhong, *Energy Environ. Sci.*, **2024**,*17*, 4670-4680.

[6] J. Lee, S. Bae, Y. Hsieh, N. De Marco, M. Wang, P. Sun and Y. Yang, Chem, **2017**, *3*, 290-302.
